# Revealing rhythm categorization in human brain activity

**DOI:** 10.1126/sciadv.adu9838

**Published:** 2025-07-30

**Authors:** Francesca M. Barbero, Tomas Lenc, Nori Jacoby, Rainer Polak, Manuel Varlet, Sylvie Nozaradan

**Affiliations:** ^1^ Institute of Neuroscience (IoNS), University of Louvain (UCLouvain), Brussels, Belgium.; ^2^ Basque Center on Cognition, Brain and Language (BCBL), Donostia-San Sebastian, Spain.; ^3^ Computational Auditory Perception Group, Max Planck Institute for Empirical Aesthetics, Grüneburgweg 14, 60322 Frankfurt am Main, Germany.; ^4^ Department of Psychology, Cornell University, Ithaca, NY 14853, USA.; ^5^ RITMO Centre for Interdisciplinary Studies in Rhythm, Time and Motion, University of Oslo, Oslo, Norway.; ^6^ Department of Musicology, University of Oslo, Oslo, Norway.; ^7^ The MARCS Institute for Brain, Behaviour & Development, Western Sydney University, Sydney, Australia.; ^8^ International Laboratory for Brain, Music and Sound Research (BRAMS), Montreal, Canada.

## Abstract

Humans across cultures show an outstanding capacity to perceive, learn, and produce musical rhythms. These skills rely on mapping the infinite space of possible rhythmic sensory inputs onto a finite set of internal rhythm categories. What is the nature of the brain processes underlying rhythm categorization? We used electroencephalography to measure brain activity as human participants listened to a continuum of rhythmic sequences characterized by repeating patterns of two interonset intervals. Using frequency and representational similarity analyses, we show that brain activity does not merely track the temporal structure of rhythmic inputs but, instead, produces categorical representation of rhythms. These neural rhythm categories arise automatically, independent of any motor- or timing-related tasks, yet exhibit strong similarity with categorization observed in overt behavior. Together, these results and methodological advances constitute a critical step toward understanding the biological roots and diversity of musical behaviors across cultures.

## Teaser

Human brain activity shows automatic categorization of rhythm, independent of but similar to categories produced in behavior.

## INTRODUCTION

A fundamental function of the brain is to enable adaptive behavior in an environment full of remarkably diverse, dynamic sensory signals. Specifically, although constantly stimulated with a wide range of inputs, the brain does not treat each sensory input as a novel, unique event—a process that would be overwhelming for the organism—but, instead, categorizes it (*1*–*5*). Perceptual categorization thus constitutes a core brain function, allowing the production of different responses to inputs belonging to different categories (discrimination) and, at the same time, identical responses to inputs belonging to the same category (generalization) (*6*).

A compelling illustration of this phenomenon is the central role categorization plays in human social interaction through musical rhythm (*7*–*9*). In music, rhythm usually refers to patterns of durations between successive sensory events [i.e., interonset intervals (IOIs) between successive notes or strokes of a percussion instrument]. For example, we easily recognize and reproduce the rhythm of “Jingle Bells” or the stomp-stomp-clap groove of “We Will Rock You” as a prototypical rhythm composed of three time intervals, where the first and second intervals are simply twice shorter than the third interval (i.e., “Jin-” and “gle” versus the twice-longer “Bells”; same for “stomp” and “stomp” versus the twice-longer “clap”), hence the 1:1:2 ratio code often used to describe it (audio S1). Crucially, we can easily recognize the rhythm of “Jingle Bells” even when the performed version deviates from a perfect template-like rendition of the time intervals. We are also able to discriminate this 1:1:2 rhythm from other distinctive three-interval rhythms, such as the so-called “tresillo” or “zouk” that is pervasive in many Afro-diasporic music genres over the world (*10*–*13*) (with a prototypical 3:3:2 ratio; audio S2).

Hence, rhythm categorization enables us to recognize musical rhythms, by allowing an infinite space of possible rhythmic sensory inputs to be carved up into a finite set of internal categories. Critically, the existence of rhythm categories has been corroborated empirically using a number of behavioral paradigms (*14*–*24*) and is a phenomenon consistently found across cultures (*10*). Several studies have shown that rhythmic patterns from different categories are easier to discriminate than rhythms falling into the same category (*14*, *20*, *22*). Likewise, identification of rhythms shows sharp transitions occurring near putative category boundaries rather than reflecting gradual physical changes in the stimuli (*14*, *15*). Moreover, sensorimotor synchronization, continuation, and iterative reproduction studies have provided robust evidence of biases toward certain rhythm categories regardless of effector and level of musical training (*10*, *16*–*19*, *22*–*24*). These (re)production biases have been interpreted through the concept of rhythm prototypes, which are thought to be closely tied to rhythm categories, representing specific patterns of relative durations (i.e., specific rhythmic ratios) in reference to which other rhythmic stimuli would be recognized, thus acting as a “perceptual magnet” (*25*) or “attractor” (*17*).

What are the biological processes underlying rhythm categorization? One view is that rhythm categories stem from hard-wired neurobiological predispositions constraining internal representations of rhythmic inputs. In particular, it has been proposed that rhythms corresponding to mathematically simple ratios [i.e., small integer ratios based on a grid of equal time intervals and their grouping in twos, such as the 1:1:2 rhythm of “Jingle Bells” (*18*)] should be universally privileged as a direct consequence of fundamental dynamics of neural assemblies (*26*). This view has been recently corroborated by large-scale behavioral research showing small–integer ratio categories, and particularly those based on the very smallest integers (1 and 2), to be universally present across cultures (*10*). This view also predicts small–integer ratio categories to emerge automatically at early stages of the sensory processing pathway.

However, a growing body of work points toward rhythm categorization as a plastic function, reflecting enculturation and social learning (*22*, *27*, *28*). This view is supported by converging behavioral and modeling evidence that rhythm categories are not fully predetermined by hard-wired neurobiological processes (*15*, *20*) but are shaped by culture-specific and individual experience (*10*, *18*, *22*, *24*, *28*, *29*). Relatedly, rather than emerging spontaneously at early processing stages, rhythm categorization may be subserved by a plastic network of higher-level sensory, motor, and associative cortices, whose activity is shaped by the distribution of rhythms in the perceivers’ environment (*22*, *27*, *28*). Moreover, this brain network could be selectively engaged depending on task demands (*30*), such as during sensorimotor reproduction or explicit discrimination [see (*4*, *31*–*33*)].

Therefore, clarifying the interplay between hard-wired mechanisms and culture-driven neural plasticity appears a critical step to understand how socially meaningful categories of rhythm are produced and transmitted. Yet, this endeavor has proven particularly challenging so far, due to the lack of task-independent measures to capture rhythm categorization from neural responses. More broadly, task-free measures are ultimately key to probe rhythm categorization across the lifespan, cultures, and species and address long-standing questions regarding the nature and underlying mechanisms of human rhythmic behaviors. Here, we address this gap by providing neural evidence for rhythm categorization and underlying rhythm prototypes, thus advancing a critical step beyond previous findings limited to behavioral measures (*14*–*24*). To this end, we developed a methodological approach combining (i) electrophysiology, (ii) frequency-domain analysis (*34*–*37*), and (iii) the representational similarity analysis (RSA) framework (*38*, *39*), hereafter referred to as frequency-RSA (fRSA).

Using the fRSA approach, we provide direct evidence for neural categorization of rhythm in humans. Specifically, we show that brain activity captured with surface electroencephalography (EEG) goes beyond mere tracking of acoustic temporal features of the rhythmic inputs and, instead, exhibits categorical representations. Moreover, we show that these neural rhythm categories emerge automatically, without any related explicit task, yet they are remarkably similar to the categorical structure reflected in sensorimotor reproduction of the same stimuli. Despite this automaticity, these rhythm categories are not fully explained by feedforward nonlinearities in the earliest stages of the ascending auditory pathway, as tested with a biomimetic model of auditory nerve responses. Therefore, by ruling out that the process of rhythm categorization merely reflects motor, instructional, or decisional biases, our results take a critical step forward in understanding the nature and neural pathways underlying this function fundamental to the human experience of music.

## RESULTS

**Fig. 1.**
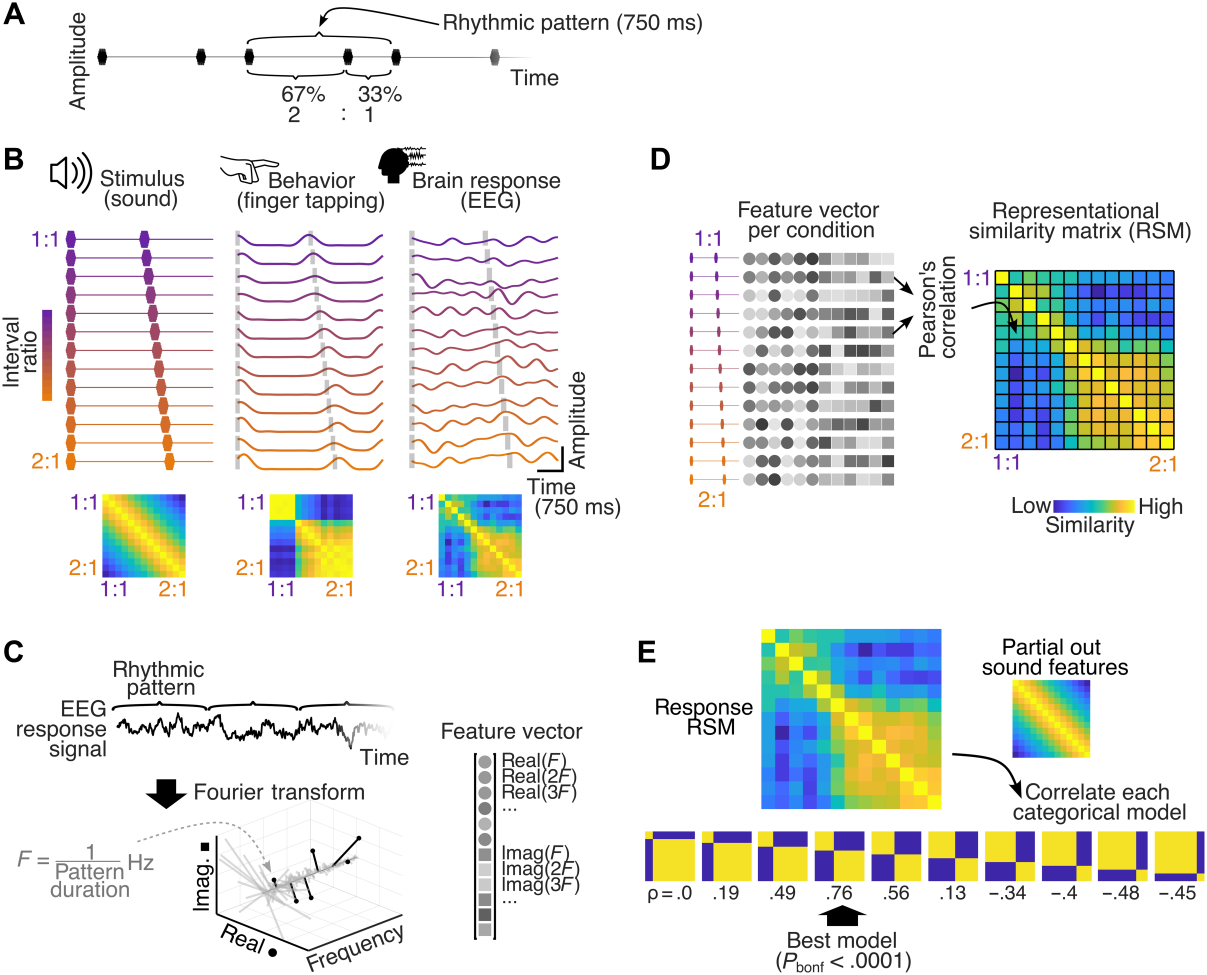
Experimental design and fRSA approach. (**A**) Example excerpt of the auditory stimulus from one condition, showing repetitions of the two-interval pattern used to construct stimulus sequences. Individual tones are indicated with black markers. One repetition of the rhythmic pattern is highlighted using brackets above the stimulus. The two constituent IOIs are indicated with brackets below the stimulus, showing the percentage of the total pattern duration spanned by each interval and the ratio of the two intervals. (**B**) Left: Time course of the two-interval pattern used to construct the stimulus sequence in each condition, with colored markers indicating tone positions. The ratio of the two constituent intervals gradually changes across the 13 conditions from 1:1 to 2:1. Below is the representational similarity matrix (RSM) depicting acoustic similarity between pairs of stimuli. Middle: Average time course of the tapping force from an example participant in each condition, with tone onsets indicated by gray bars. The tap-force RSM below was built using fRSA. Right: Trial-averaged EEG responses of an example participant and the corresponding neural RSM. (**C**) Feature extraction: the top panel shows a segment of an example EEG neural response to repetitions of the rhythmic pattern. The complex-valued spectrum of the EEG obtained with Fourier transform is shown below. The response is concentrated in a priori known frequency bins corresponding to 1/rhythmic pattern duration and harmonics (in black). Real and imaginary coefficients at response frequencies are concatenated into a feature vector shown on the right. (**D**) RSM built by correlating feature vectors across all pairs of conditions. (**E**) Fitting categorical models to the response data: The neural RSM of an example participant is correlated with each categorical model RSM (differing in the category boundary location) after removing the contribution of the acoustic model RSM. The *P* value is obtained using permutation testing.

Using scalp EEG, we recorded brain activity of healthy adult participants (*n* = 18) as they listened to different sequences, each made of a repeated rhythmic pattern (750-ms pattern duration), while carrying out a volume-change detection task unrelated to the goal of the study. Each pattern consisted of two identical tones, which, when looped, yielded a repeating sequence of two time intervals. Across conditions, we manipulated the ratio between the duration of the first and the second time interval to span the continuum of two-interval rhythms, ranging from isochrony (1:1 interval ratio) to patterns of long-short intervals with a 2:1 ratio (hereafter also expressed as ratios from 0.50 to 0.67, respectively, when dividing the first interval by the sum of the two intervals; Fig. 1, table S1, and audio S3).

We used this rhythm continuum based on previous results consistently showing that rhythms from this continuum are perceived by Western participants as two discrete categories separated by a sharp perceptual boundary (*14*, *15*, *22*). In addition, each of these categories has been characterized by a prototypical rhythmic pattern, one centered on 1:1 and the other close to (but not necessarily at) 2:1 ratio (*16*–*19*). There is abundant evidence that sensorimotor synchronization and reproduction of rhythms falling in each of these categories are distorted toward the corresponding prototype, thus broadly compatible with the concept of a perceptual attractor from dynamical systems literature (*16*, *17*) as well as a Bayesian concept of a perceptual prior (*22*, *27*). To obtain a behavioral measure of rhythm categorization, we also asked the same participants to tap their finger on a response sensor in the best possible synchrony with the stimuli (in blocks separate from the EEG recordings).

### Behavioral evidence for rhythm categorization

**Fig. 2.**
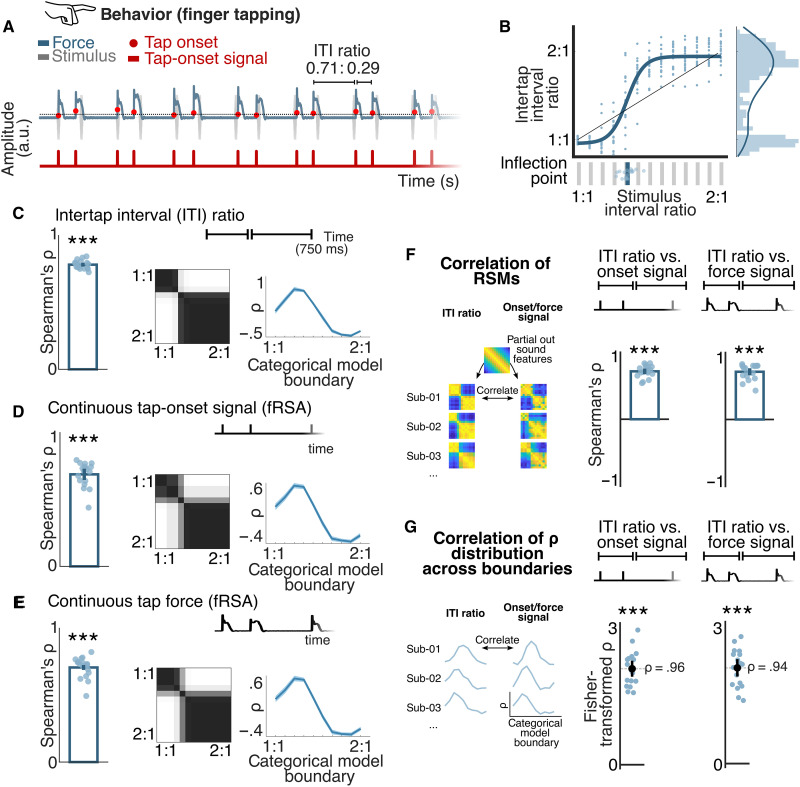
Behavioral responses show consistent categorization across participants and tapping signals. (**A**) Example segment of a continuous tapping force signal with superimposed tap onsets (top) and the corresponding continuous tap-onset signal (bottom). Two ITIs and their ratio within an example repetition of the rhythmic pattern are highlighted with brackets. a.u., arbitrary units. (**B**) Average ITI ratio as a function of stimulus IOI ratio. Responses from individual participants are shown as circles. The black diagonal line corresponds to identical ITI and IOI ratios (undistorted reproduction). The blue line depicts a sigmoid function fit to data pooled across all participants, with its 50% threshold shown as a vertical blue line in the panel below (estimates for individual participants are plotted as circles). The histogram and density of the pooled distribution of ITI ratios are shown on the right. (**C**) Left: Correlation of the ITI RSM with the best-fitting categorical model RSM (individual participants shown as circles). Middle: Overlay of best-fitting categorical model RSMs across participants. Right: Distribution of correlations across all possible categorical models differing in category boundary location (group average). (**D**) Same as (C), for the continuous tap-onset signal. (**E**) Same as (C), for the continuous force signal. (**F**) Participant-wise correlation of the RSM based on ITI ratios and continuous tap onset (left) and ITI ratios and continuous tap force (right). Individual correlation coefficients are shown as circles (filled circles indicate significance at the individual level). (**G**) Fisher-transformed correlation between the distribution of correlations across all possible categorical models obtained for the ITI RSM and the tap-onset RSM (left) or the tap-force RSM (right). In all panels, error bars and shaded regions represent a 95% CI, and asterisks indicate a significant permutation test at the group level (Bonferroni corrected, ***P < 0.001).

First, we tested whether rhythm categorization was evident in the behavior by analyzing the intertap intervals (ITIs) produced in each condition when participants were instructed to tap the finger in synchrony with the rhythmic inputs (Fig. 2A). To this aim, we calculated the ratio of the first over the sum of the first and second ITI separately for each of the 90 (30 per sequence × 3 trials) repetitions of the 750-ms-long rhythmic pattern. After cleaning (see Materials and Methods), ITI ratios were averaged across rhythm repetitions separately for each condition and participant (mean number of averaged ITI ratios per condition = 64, range = 16 to 84). In line with previous studies (*17*, *19*, *21*, *22*), the produced ITI ratios did not faithfully reproduce the ratios of the corresponding stimuli (Fig. 2B). Instead, they revealed two categories, whereby the tapping near the 1:1 and 2:1 conditions (which can also be expressed as 0.50 and 0.67) exhibited a ratio close to 1:1 and 2:1, respectively. Indeed, the distribution of ITI ratios across conditions was significantly better explained by a sigmoidal compared to a linear model (*P* = 0.002, Wilcoxon rank sum test comparing leave-one-participant-out cross-validated *R*^2^). Moreover, the mean inflection point of the sigmoidal fit across participants did not overlap with the mathematical midpoint of the ratio continuum tested in the current study (i.e., 0.58) but corresponded instead to a median ratio of 0.56 [bootstrapped 95% confidence interval (CI) = 0.555 to 0.563, computed by resampling participants with replacement], that is, relatively closer to the 1:1 ratio edge of the continuum, thus revealing a smaller and a bigger category (*14*). The two categories were also apparent in the distribution of produced ITI ratios collapsed across all conditions and participants that was significantly different from a uniform distribution [χ^2^(12) = 113.51, *P* < 0.0001]. Taken together, the obtained results corroborated a well-established effect in the literature (*15*, *18*, *22*).

After capturing the categorical structure using a standard approach by directly modeling the produced ITI ratios, we asked whether the same categories would emerge when analyzing the similarity, rather than the values of the produced ratios, across conditions. To this end, we built a representational similarity matrix (RSM) for each participant, based on the absolute differences in the average ITI ratios produced across all pairs of conditions. Each individual ITI RSM was then compared against several theoretical models of categorization differing in the position of the category boundary (yielding 10 distinct theoretical models in total; Fig. 1E), controlling for the variance explained by acoustic features via partial correlation (Fig. 1E and see Materials and Methods). The best-correlated categorical model was selected for each participant, and significance was evaluated using a permutation test in which RSM entries were randomly shuffled. As shown in Fig. 2C, the ITI RSM of each participant was significantly correlated with a categorical model (*P*_bonf_s < 0.05; Bonferroni corrected for the 10 tested theoretical models; see also fig. S1). The group-level correlation, based on the average of individual coefficients, confirmed a strong and reliable effect (permutation test, ρ = 0.78, *P*_bonf_ = 0.001). The best-fitting categorical models were consistent across individuals, and the observed category boundary (median ratio = 0.55 and bootstrapped 95% CI = 0.55 to 0.56) was compatible with the one identified with ITI ratio analysis.

### Revealing rhythm categorization from continuous signals

Next, we extended the ITI-based findings by testing whether a similar categorical structure could be observed when representing the series of tap onsets as a continuous signal. This constitutes a pivotal shift from the analysis of time intervals defined by discrete temporal markers toward the analysis of continuous data where the identification of temporal markers may be less straightforward (e.g., as in surface EEG). To this end, we applied the fRSA approach to the tapping responses represented as continuous time-varying signals with a unit impulse at the onset time of each executed tap (see Fig. 2A). Notably, and in contrast with ITI ratios, such representation is sensitive to the stability of the produced intervals over time and trials, thus constituting a more comprehensive measure to capture categorization in continuous signals.

We used frequency-domain analysis to isolate and characterize the time course of the response signal in each condition (Fig. 1C). For each participant and condition, the spectrum of trial-averaged continuous tap-onset signals was obtained using Fourier transform. A critical advantage of using frequency-domain analysis in the current experimental design is that it enables isolating responses to the repeated rhythmic pattern from the recorded continuous signals with high objectivity and signal-to-noise ratio (*34*–*37*). Indeed, in line with well-established principles of frequency tagging (*34*, *40*, *41*), any response that consistently repeats across repetitions of the periodically presented rhythmic pattern is effectively described by a set of complex Fourier coefficients at the frequency of the pattern repetition and harmonics.

As expected, based on this principle, the obtained spectra averaged across all participants and conditions exhibited peaks of magnitude at frequencies corresponding to the repetition rate of the rhythmic pattern and harmonics (i.e., *f* = 1/0.75 s = 1.33 Hz, 2*f* = 2.66 Hz, and so on; fig. S2). The magnitude of the response at these a priori determined frequencies was significantly above the local noise baseline (*42*–*44*) for harmonics up to 16 Hz (*z* > 3.09, i.e., *P* < 0.001, one-tailed, signal > noise), which were thus further considered as frequencies of interest (fig. S3). Responses at these frequencies of interest were used to build an RSM for each participant. First, the real and imaginary Fourier coefficients obtained at harmonics up to 16 Hz were concatenated to form a feature vector for each condition (Fig. 1C), thus yielding a complete yet low-dimensional description of response time course (including amplitude and timing information). Subsequently, an RSM was built to reflect the similarity of feature vectors across conditions (Fig. 1D).

There was a significant correlation between tap-onset RSMs and categorical models at the group level (permutation test, ρ = 0.69, *P*_bonf_ = 0.001), as well as at the individual level in all participants (permutation test, *P*_bonf_s < 0.05; Fig. 2D and fig. S1). As for the results obtained with the RSA applied to ITI ratios, there was a marked cross-participant consistency in the category boundary that best explained the RSMs obtained with fRSA applied to the continuous tap-onset signals (median boundary ratio = 0.56 and bootstrapped 95% CI = 0.55 to 0.56; Fig. 2D).

The correlation between the RSM and the best-fitting categorical model selected for each participant was significantly smaller when considering the tap-onset signals rather than ITIs [paired *t* test, *P* < 0.001, Bayes factors (BF_10_) = 76]. This was not surprising given that frequency-domain analysis of tap-onset signals is more conservative as compared to ITI-ratio analysis, which comprised (i) a cleaning step whereby responses to pattern repetitions where the participant was not complying with the synchronization task were discarded from further analyses and (ii) an inherent time-warping of the response to each pattern repetition that corrects for tempo drifts (as the absolute durations of the two ITIs produced on each pattern repetition are normalized by taking a ratio). Nonetheless, there was a remarkable similarity between the RSMs obtained from ITIs and from the tap-onset signals (group-level permutation test, ρ = 0.72, *P* = 0.0001; Fig. 2F). Moreover, the relative distribution of correlation coefficients across theoretical models with different locations of the category boundary was remarkably similar between the two analyses (mean ρ across participants = 0.96, one-sample *t* test against zero, *P* < 0.0001; Fig. 2G). These findings thus present a key validation that fRSA can reveal categorization encoded in the temporal structure of a response without relying on the extraction of discrete temporal markers.

To further demonstrate the robustness of fRSA in revealing rhythm categories from continuous data, we applied the method to continuous force signals obtained directly from the tapping sensor. In addition to the mere temporal arrangement of the taps, the time course of the force signal also depends on their relative accentuation and overall kinematics of their execution, thus potentially offering yet another dimension that could reflect categorization of rhythmic inputs. Inspecting the grand-average magnitude spectrum of the tapping force revealed peaks at the rate of the rhythmic pattern repetition up to 16 Hz (*z* > 3.09, i.e., *P* < 0.001, one-tailed, signal > noise; figs. S2 and S3). We thus used the real and imaginary Fourier coefficients at these frequencies of interest to build a tap-force RSM for each participant.

**Fig. 3.**
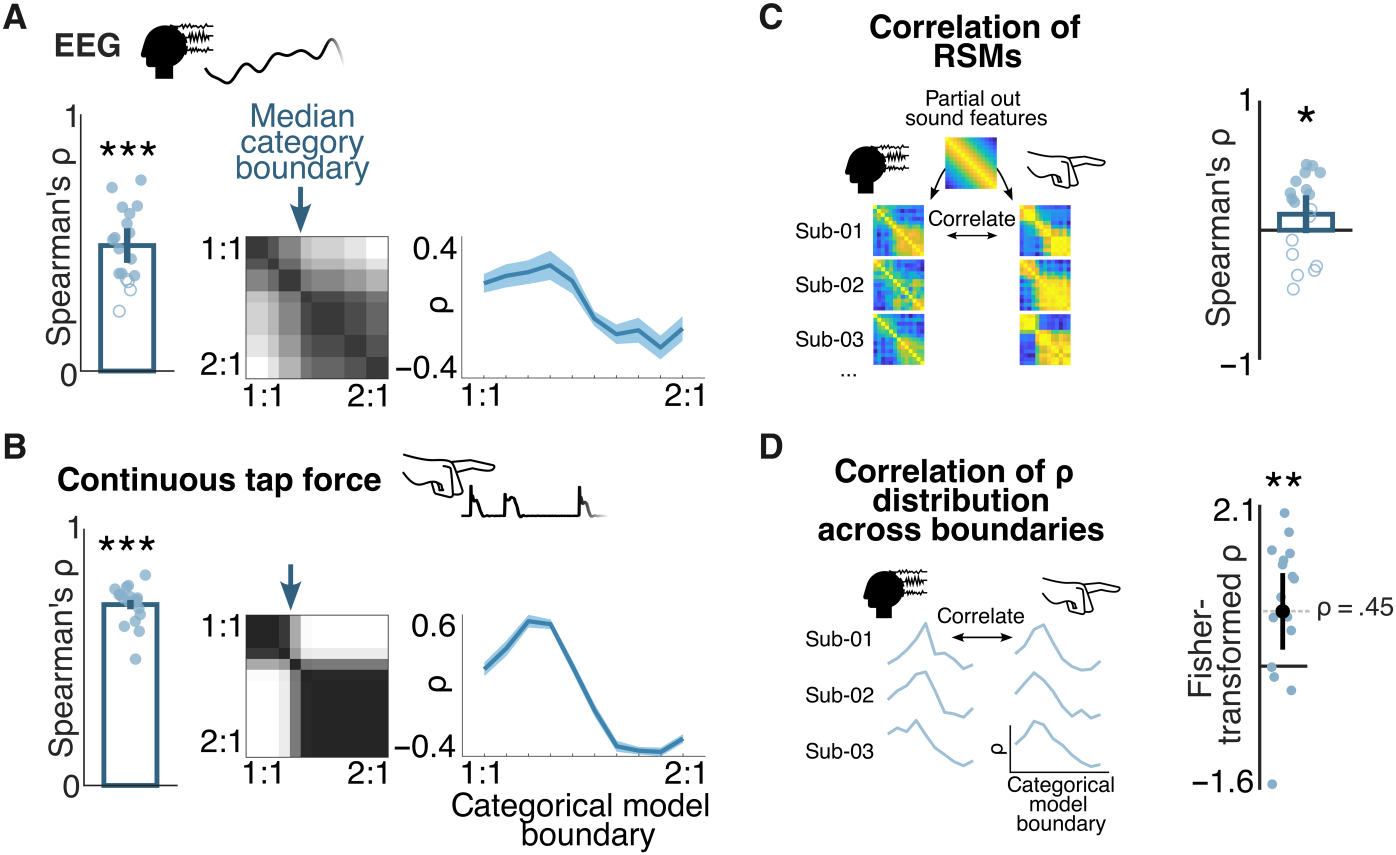
Convergent evidence for rhythm categorization from neural and behavioral responses, as captured with fRSA. (**A**) fRSA analysis of EEG responses (responses from 64 channels, considering frequencies up to 8 Hz). Left: Blue circles show Spearman’s correlation of the EEG response RSMs with the best-fitting categorical model, obtained separately for each participant. Filled circles indicate a significant permutation test at the individual participant level, and asterisks indicate a significant permutation test at the group level (Bonferroni corrected, ***P < 0.001). Middle: Overlay of best-fitting significant categorical models across participants, with median category boundary indicated with a blue arrow. Right: Distribution of correlations across all possible categorical models differing in the location of the category boundary (averaged across participants, shaded regions indicate 95% CI). (**B**) Same as (A), but applied to continuous tapping force (considering frequencies up to 16 Hz). (**C**) Participant-wise correlation of the neural RSM and the corresponding tap-force RSM. The shared information driven by stimulus features was removed by partialing out the acoustic model RSM. Data from individual participants are shown as blue circles (filled circle indicates a significant permutation test at the individual participant level). Error bars represent a 95% CI. Asterisks indicate a significant permutation test at the group level (*P < 0.05). (**D**) Fisher-transformed correlation between the distribution of correlations across all possible categorical models obtained for the neural RSM and the tap-force RSM. Blue circles correspond to individual participants, error bars represent a 95% CI, and asterisks indicate a significant *t* test against zero (**P < 0.01).

At the group level, the obtained tap-force RSMs showed clear correspondence with a categorical representational structure (permutation test, ρ = 0.71, *P*_bonf_ = 0.001), which was also significant in each individual participant (permutation test, *P*_bonf_s < 0.05; Figs. 2E and 3B and fig. S1), and consistent across participants (median boundary ratio = 0.55 and bootstrapped 95% CI = 0.55 to 0.56). The average correlation with a categorical model was significantly smaller for RSMs built by applying fRSA to the force signal, as compared to the RSMs obtained from ITI ratios (paired *t* test, *P* < 0.001, BF_10_ = 70). Yet, similarly to the results obtained with fRSA of tap-onset signals reported above, the categorical structure captured by the tap-force fRSA was markedly consistent with the one identified using ITI ratios. Specifically, the corresponding RSMs obtained by each method highly correlated within participants (group-level permutation test, ρ = 0.72, *P* = 0.0001; Fig. 2F) and revealed a similar position of the category boundary (mean ρ across participants = 0.94, one-sample *t* test against zero, *P* < 0.0001; Fig. 2G).

These results thus highlight the fRSA approach as a conservative yet sensitive method to capture categorical structures from continuous smooth signals beyond relying on discrete homogenous temporal markers. Notably, these results also indicate that including additional information about the way each tap movement is executed (here, including both tap-onset and force information) yields representational structures overall consistent with those obtained with tap timing alone. In line with prior work (*16*), these results provide further support to the view that the timing of motor responses elicited during a sensory-motor synchronization task is the key dimension that reflects rhythm categorization in human adults.

Overall, our analyses of the tapping data (i) confirm that, in accordance with previous studies, the set of two-interval rhythms used in the current study elicited internal representation of two separable rhythm categories and (ii) validate the fRSA method as a robust and sensitive tool to identify rhythm categories using continuous time-varying response signals beyond the need to extract discrete temporal markers. These constitute critical prerequisites for proceeding to the analysis of EEG data.

### Human brain activity shows automatic categorization of rhythm

We recorded brain activity using EEG as participants listened to the rhythmic stimuli without performing any overt movement. As for the tapping, trial-averaged preprocessed EEG responses were transformed into the frequency domain using Fourier transform. Here, we only considered frequencies of interest up to 8 Hz, as this frequency cutoff captured all significant responses at consecutive harmonics of the rhythm repetition rate as observed in the grand-average EEG magnitude spectrum (i.e., first six harmonics of the rhythmic pattern repetition; *z* > 3.09, i.e., *P* < 0.001, one-tailed, signal > noise; figs. S2 and S3). Higher frequencies were also excluded to avoid distortion of the EEG spectra by the alpha activity artifact (approximately between 8 and 12 Hz) (*45*–*47*).

For each participant, we built a neural RSM based on the similarity of real and imaginary Fourier coefficients at the frequencies of interest concatenated across all 64 EEG channels, thus accounting for any individual differences in response topography. The neural RSMs exhibited significant categorical structure at the group level (permutation test, ρ = 0.49, *P*_bonf_ = 0.001; Fig. 3A). Moreover, we were able to observe significant correlation with a categorical model at the individual level in most participants (14 of 18; permutation test, *P*_bonf_s < 0.05; Fig. 3A, fig. S1, and table S2).

Notably, we observed comparable evidence of neural categorization when limiting the analysis to responses averaged across nine frontocentral channels (fig. S4). This pool of channels was selected based on the fact that they have been shown to consistently capture EEG responses to repeating acoustic rhythms across previous studies (*36*, *48*–*50*). Analogously, these channels also showed the highest overall response magnitude averaged across all participants and conditions in the current study (fig. S3). This result suggests that accounting for individual differences in response topography is not critical to reveal categorical encoding of auditory rhythmic inputs. Instead, it appears that spatiotemporal dynamics captured in the time course of brain activity at a single scalp location (with high signal-to-noise ratio) are sufficient to efficiently reveal the representational geometry relevant for rhythm categorization.

Finally, having acknowledged the theoretical advantages of frequency-domain analysis, we asked whether a time-domain analysis would yield comparable results. Notably, we first low-pass filtered the EEG response at 10 Hz to capture its frequency range as determined from the magnitude spectrum above (thus still partly capitalizing on the ability to estimate response bandwidth in the frequency domain). RSMs were then built by correlating the average time course of the response over the duration of the rhythmic pattern across all pairs of conditions. We observed strong evidence of neural categorization (group-level permutation test, ρ = 0.48, *P*_bonf_ = 0.001; significant permutation test in 12 of 18 participants, *P*_bonf_s < 0.05; fig. S5A), corroborating the results of the frequency-domain analysis. Indeed, the neural RSMs built from the frequency domain and time domain (both based on the average response across nine frontocentral channels) were highly correlated within participants beyond the shared information driven by stimulus features (group-level permutation test, average ρ = 0.95, *P* = 0.0001; fig. S5B). These convergent results are expected, as time domain averaging acts as a comb filter that effectively isolates frequencies corresponding to pattern repetition and harmonics, which is a fundamental rationale underlying fRSA. Yet, the frequency-domain analysis yielded larger correlation with the best categorical model as compared to the time-domain analysis ( $\rho_{\text{freq}.}$ − $\rho_{\text{time}}$ = 0.02, *P*_bonf_ = 0.04; fig. S5C), thus further underscoring the advantages of our approach. Taken together, these results provide evidence for neural categorization of rhythm, whereby neural activity elicited without related temporal task or overt movement represents the acoustic continuum of two-interval rhythms as two distinct categories.

In our fRSA analysis of the EEG responses reported above, we controlled for potential contribution of low-level tracking of the sensory input to the obtained categorical structure by partialing out the acoustic RSM (Fig. 1E). However, it remains unclear whether the observed transformation from acoustic features toward a categorical representation of rhythm reflects a higher-level, possibly cortical, neural processing and to what extent it could be shaped by low-level nonlinearities of subcortical neurons from the ascending auditory pathway. We explored this possibility by taking advantage of a well-established biologically plausible model of the auditory periphery to simulate responses elicited by our acoustic stimuli in a set of auditory nerve fibers (*51*).

The model provided faithful simulation of physiological processes associated with cochlear nonlinearities, inner hair cell transduction process, the synapse between the hair cell and the auditory nerve, and the associated firing rate adaptation. For each condition, we simulated the time course of instantaneous firing rate in the auditory nerve elicited by the corresponding rhythmic sequence (fig. S6A). An auditory nerve RSM was then obtained from these simulated responses by applying the same fRSA analysis as for the EEG responses above.

Crucially, the auditory nerve RSM closely resembled the acoustic RSM (fig. S6B), indicating that the modeled early subcortical representations mainly followed the temporal structure of the stimulus sequences. More specifically, the obtained auditory nerve RSM did not show significant rhythm categorization (no significant correlation with a categorical model; permutation test, ρ = 0.33, *P*_bonf_ = 0.18), in contrast with the EEG responses. Indeed, when accounting for structure explained by the auditory nerve RSM using partial correlation, the EEG responses still showed categorical representation of rhythm significant both at the group level (permutation test, ρ = 0.51, *P*_bonf_ = 0.001) and at the individual level in 16 of the 18 participants (permutation test, *P*_bonf_s < 0.05). Together, these results suggest that neural categorization of rhythm does not likely arise as a product of nonlinear transformations occurring at the earliest, peripheral stage of the ascending auditory pathway.

### Convergence across behavioral and neural categorization of rhythm

Our results indicate that both behavioral and neural responses to the rhythmic inputs do not simply reflect acoustic features but, instead, exhibit representational geometries consistent with the existence of two distinct rhythm categories, a smaller one spanning ratios from about 0.50 to 0.55 (1:1 to 1.2:1) and another, bigger one spanning from 0.56 to 0.67 (1.3:1 to 2:1). However, is there a correspondence between the categories reflected in the brain and behavior?

To answer this question, we first assessed the overlap between the representations measured from the brain and behavior of each participant, by correlating the neural RSM (considering frequencies up to 8 Hz and all 64 channels) with the corresponding tap-force RSM (considering frequencies up to 16 Hz), while partialing out the shared similarity structure driven by the acoustic stimulus. The neural RSMs significantly correlated with the tap-force RSMs at the group level (permutation test, ρ = 0.12, *P* = 0.03), and this correlation was significant in 10 of 18 participants (Fig. 3C). Similar results were obtained when correlating neural RSMs with tap-onset RSMs (group-level permutation test, ρ = 0.16, *P* = 0.007) or tap-ITI RSMs (group-level permutation test, ρ = 0.21, *P* = 0.003). These results suggest that the neural and behavioral responses share similar representational geometries beyond faithful encoding of acoustic features.

Next, we examined the location of the categorical boundary separating the two rhythm categories observed in the EEG and tap-force RSMs. Across participants, the location of this boundary in the best-fitting categorical model was remarkably similar for the EEG (median boundary ratio = 0.56 and bootstrapped 95% CI = 0.54 to 0.57; Fig. 3A) and the tap-force responses (median boundary ratio = 0.55 and bootstrapped 95% CI = 0.55 to 0.56; Fig. 3B).

This result was further corroborated by considering all theoretical models of categorization differing in the position of the category boundary, rather than a single best-fitting model. Indeed, the distribution of correlation coefficients obtained across all categorical models separately for each participant showed marked similarity between the neural and behavioral (tap-force) responses (mean ρ across participants = 0.45, one-sample *t* test against zero, *P* = 0.01; Fig. 3D). Together, these observations highlight the consistency across neural and behavioral representations of the two-interval rhythms tested in the current study.

### Identifying the underlying prototypes

Using fRSA, our results provided evidence for categorical structure in continuous response signals. Building on this, we sought to further characterize the specific form of the responses making up each identified category. Indeed, the categorical structure observed here could have been driven by any feature of the response that systematically differed across the two categories (i.e., discriminated) and remained consistent for conditions within each category (i.e., generalized). Thus, the relevant property could have been, for example, the response amplitude at an arbitrary yet systematic latency. Alternatively, categorization may have emerged from the temporal profile of the response, whereby a consistent trajectory would repeat according to a recurring pattern of time intervals with a given ratio.

While the latter hypothesis was supported by the analysis of tapping responses above, characterizing the relevant features of EEG responses requires a more general approach that does not rely on the presence of homogeneous discrete temporal events. To this end, we carried out an exploratory analysis of response trajectory in each condition based on the similarity with a set of prototypical temporal templates.

As shown in fig. S7, the prototypes consisted of continuous signals made of unit impulses arranged over time to create a repeating pattern of two time intervals (pattern duration was set to 750 ms, as for the stimuli). Across prototypes, ratios of the constituent intervals were equally spaced between 0.50, i.e., the 1:1 edge of the condition continuum tested here, and 0.84, i.e., beyond the 2:1 ratio (0.67) corresponding to the other edge of the condition continuum, yielding a total of 76 prototype signals. This set of prototypes thus corresponded to a wide and fine gradient of two-interval ratios [note that ratios from 0.84 up to 0.99 were not included in this set of prototypes as these ratios would result in shortest interval durations shorter than 127.5 ms, thus likely reaching motor constraints for one-to-one sensorimotor synchronization of finger tapping in nonmusicians (*52*)].

Next, we assessed which prototype was the most similar to the response in each condition. Critically, this question cannot be answered by directly comparing the time course of the prototype and the response, as this would impose unnecessary assumptions about the shape and phase lag of the repeated trajectory [for further discussion, see (*40*)]. Likewise, taking an approach similar to the fRSA above and assessing prototype-response similarity based on complex Fourier coefficients could be misleading, given the mathematical equivalence between a time-domain signal and its complex Fourier transform. Instead, the temporal structure of each prototype and response signal, that is, the profile of (a)symmetries in their time course, is well captured by taking the distribution of magnitudes across the frequencies of interest corresponding to the rate of rhythmic pattern repetition and harmonics [see also (*53*); fig. S7].

As a first step, we confirmed that this profile of (a)symmetries was compatible with the categories we have identified in the analysis of complex Fourier coefficients. To this aim, we compared the neural RSMs based on magnitudes at the frequencies of interest (up to 8 Hz at all 64 channels) to the corresponding RSMs built by considering the real and imaginary Fourier coefficients used in the fRSA above. While accounting for the acoustic RSM, we observed significant partial correlation between the magnitude and complex RSMs at the group level (permutation test, ρ = 0.46, *P* = 0.0001) and in 15 of 18 individual participants. A similar result was observed for the continuous tap-force responses (considering frequencies up to 16 Hz), with significant correlation at the group level (*P* = 0.0001) and in all individual participants.

Having established that the categorical structure of the responses was well captured in the magnitude spectrum, we moved to testing which prototype offered the best characterization of the response in each condition. To this end, we correlated the vector of magnitudes at the frequencies of interest taken from the spectrum of each prototype with the same vector obtained from the magnitude spectrum of the continuous response signal (either tapping force or EEG) averaged across all participants, separately for each condition. We used the grand average instead of individual participants’ spectra to further improve the signal-to-noise ratio (particularly for the EEG responses) in view of optimizing the identification of potential underlying prototypes. The statistical distribution of the maximally correlated prototype in each condition was estimated by bootstrapping (i.e., by repeatedly building the grand-average spectrum from a resampled pool of participants; see Materials and Methods and fig. S7).

**Fig. 4.**
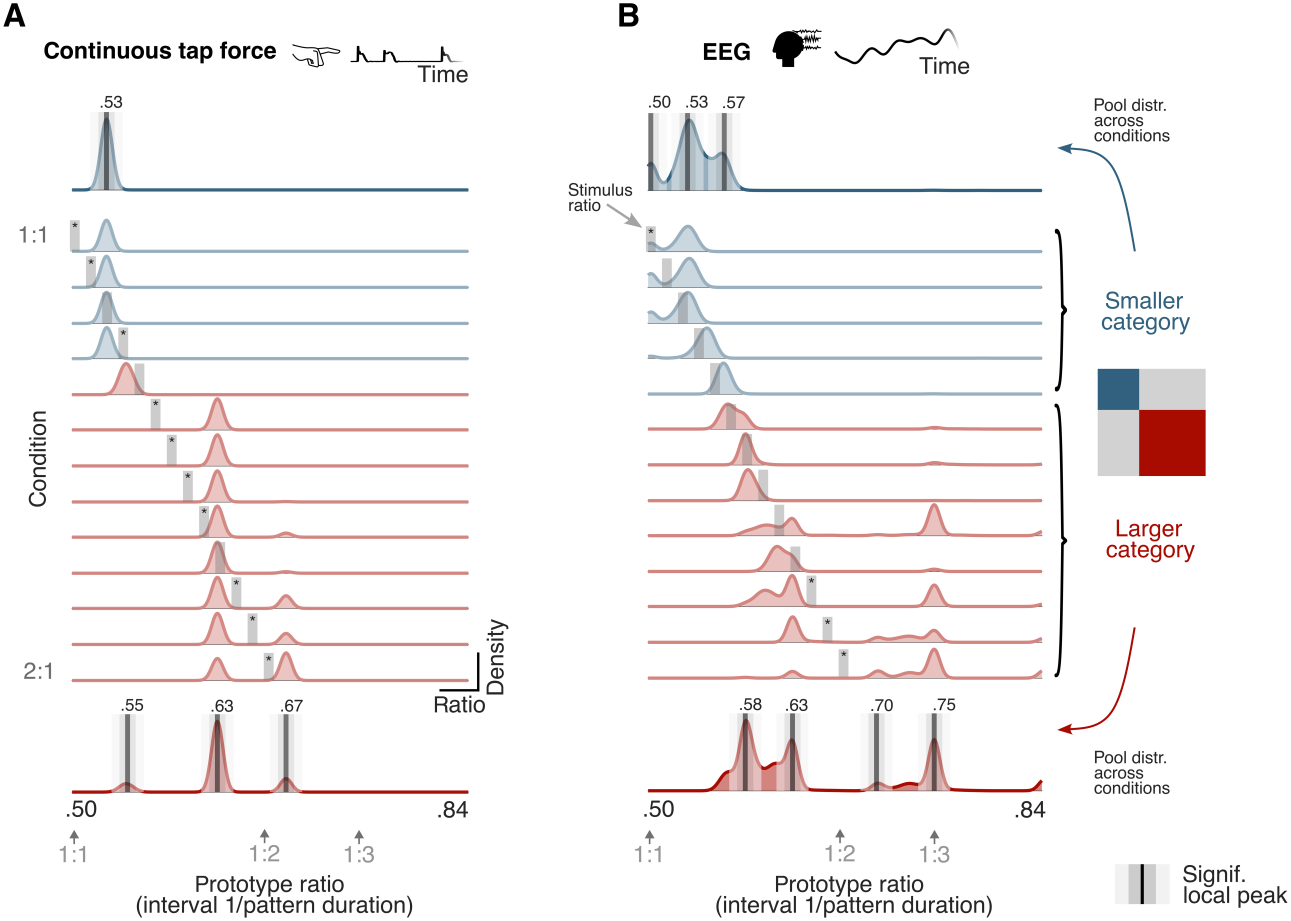
Analysis of similarity with two-interval prototypes: Identified prototypes deviate from the mathematically simplest ratios in both neural and behavioral responses. (**A**) Bootstrapped distribution of maximally correlated prototypes for the continuous tap-force responses (considering frequencies up to 16 Hz). Each row shows estimated density (*y* axis) as a function of prototype interval ratio (*x* axis; 76 prototype ratios tested) for one condition. Conditions corresponding to the smaller and bigger category identified in the fRSA analysis are shown in blue and red, respectively. The vertical gray line in each row marks the stimulus ratio in the corresponding condition. Asterisks indicate conditions with significantly greater distribution density outside versus inside a narrow window centered on the stimulus ratio. A distribution collapsed across the conditions within each category is shown on the top and bottom, respectively. Positions of significant local density peaks are depicted with vertical black lines, flanked by gray rectangles indicating snippets of the distribution that were used to quantify prominence of the particular peak (calculated as density inside the darker rectangle minus the density inside the lighter rectangle). Gray arrows below the plot indicate positions of small integer ratios on the *x* axis. (**B**) Same as (A), but for EEG responses (based on a pool of nine frontocentral channels and frequencies up to 8 Hz; note that the median category boundary estimated for the EEG responses is shifted one condition toward 2:1, as compared to the tapping responses).

For the continuous tap-force signals, the identified prototypes were not gradually changing across conditions, as would be expected if the response was following the temporal structure of the corresponding auditory stimuli (Fig. 4A). Instead, the distribution of maximally correlated prototypes was significantly concentrated away from the stimulus ratio in 11 of the 13 conditions (bootstrap test, *P*_bonf_s < 0.05).

We then assessed whether responses within each of the categories identified by the fRSA approach detailed above showed high similarity to particular prototypes. To this aim, we created marginal distributions by collapsing the distribution of maximally correlated prototypes across conditions, separately for the smaller and bigger categories. Local peaks in these marginal distributions were identified by sliding a narrow window through the prototype continuum and quantifying whether the distribution inside the window was higher than in its local neighborhood (*54*, *55*) (see Materials and Methods and fig. S7).

On the one hand, in the four conditions corresponding to the smaller category (i.e., between stimulus ratios 0.50 and 0.55), the distribution of prototypes maximally correlated with the tapping response peaked at a ratio near 0.53 (*P*_bonf_ = 0.01). On the other hand, for the nine conditions corresponding to the bigger category (i.e., stimulus ratios between 0.56 and 0.67), the tapping seemed to be best characterized by a prototype with ratio near 0.63 (*P*_bonf_ = 0.01), with smaller but significant peaks appearing near 0.67 (*P*_bonf_ = 0.01) and 0.55 (*P*_bonf_ = 0.01, yet limited to a single condition).

Together, these observations point toward underlying prototypes that would not align with small, mathematically simple, integer ratios (i.e., 0.50 and 0.67, corresponding to 1:1 and 2:1, respectively). Instead, the participants seemed to soften or sharpen the produced interval ratio in a way broadly consistent with observations from other behavioral studies (*16*–*18*).

Next, we assessed which prototypes were the most similar to the neural responses. The observed distribution of prototypes maximally correlated with the EEG responses appeared to partly follow the interval ratios of the rhythmic patterns presented in each condition (Fig. 4B). This is not surprising, as the measured EEG response is expected to reflect a mixture of stimulus features and categorization, which are less efficiently disentangled here as compared to the fRSA analysis where the contribution of stimulus features is statistically controlled using partial correlations (i.e., by partialing out the acoustic RSM). Crucially, the EEG response appeared to go beyond simple replication of the stimulus interval ratios, as suggested by significantly higher density of maximally correlated prototypes concentrated off rather than on the stimulus ratio in 4 out of 13 conditions (bootstrap test, *P*_bonf_s < 0.05). Instead, high similarity tended to cluster near a small set of prototypes, which were evident after collapsing the distribution of maximally correlated prototypes across conditions separately for each category identified with fRSA (note that for EEG, the median category boundary across participants fell between conditions 5 and 6, that is, slightly closer to the 2:1 condition as compared to the tapping force responses).

On the one hand, there seemed to be prominent similarity with prototypes corresponding to small integer ratios, indicated by significant peaks near 0.50 (i.e., isochrony; *P*_bonf_ = 0.02) or 0.75 (i.e., one interval three times longer than the other interval; *P*_bonf_ = 0.02). On the other hand, the structure of the EEG responses also appeared compatible with more complex interval ratios, including, for example, 0.53 (*P*_bonf_ = 0.02) and 0.63 (*P*_bonf_ = 0.02). Moreover, the neural categorical structure appeared to be somewhat more differentiated than the behavioral one. First, a prototype emerged at 0.58 (approximately corresponding to 4:3; *P*_bonf_ = 0.02). Second, the bias toward the prototype at 0.75, which lies outside the range of the stimuli as presented here [0.50 to 0.67 (1:1 to 1:2)], was not observed in the tapping. This prototype could reflect resonant frequencies emerging from fundamental oscillatory dynamics of neural assemblies (*26*). These observations thus lend support to the view that neural activity elicited by rhythmic inputs may reflect a combination of acoustic features and internal biases that shape the representation toward a small set of prototypical rhythmic templates that do not systematically correspond to the mathematically simplest ratios.

## DISCUSSION

The current study provides direct evidence for neural categorization of rhythm in humans. Specifically, we show that brain responses to rhythmic patterns do not merely reflect the physical temporal structure of the acoustic input. Rather, the structure of the neural responses across conditions is compatible with the existence of two distinct rhythm categories, consistent with behavioral measures from the same participants and in line with a large body of prior behavioral work (*14*–*23*). Moreover, the fact that neural categorization of rhythm emerges even in the absence of an explicit behavioral timing task indicates a largely automatic process.

Thus far, research on rhythm categorization has been restricted to behavioral measures, due to the lack of a method allowing rhythm categorization to be captured from neural data. Our results extend previous findings from discrimination and sensorimotor synchronization studies (*10*, *14*, *22*), by showing that the brain automatically maps the continuous space of two-interval rhythms onto discrete rhythm categories also in the absence of a motor or timing-related task. Crucially, this observation adds to the broad evidence arguing against the view that rhythm categorization arises in motion production from movement kinematics rather than from perceptual representations (*56*). Indeed, prior work has revealed comparable rhythm categorization in sensorimotor tasks involving various modes of production [unimanual (*19*, *21*) and bimanual (*57*) tapping and verbal reproduction (*22*)]. Yet, beyond movement kinematic properties specific to each effector, different modes of movement executions could still, in principle, reflect constraints of higher-level, central motor control rather than perceptual representations (*58*). However, without denying a role of movement in rhythm perception in general, a critical contribution of central motor control to rhythm categorization seems unlikely given robust evidence for rhythm categorization in perceptual discrimination tasks (*14*, *20*, *22*).

Here, we move a critical step beyond behavioral studies by showing here that profiles of discrimination/generalization compatible with perceptual categorization can emerge from neural activity even without engagement in related explicit judgement tasks that may be sensitive to decisional and cognitive factors potentially driven by task demands (*4*, *33*). Our results thus reinforce the view of rhythm categorization as a largely automatic function of the human brain. The notion that this function might not be under volitional control is also compatible with previous observations whereby even highly trained musicians under strict instructions cannot avoid categorical distortions in sensorimotor reproduction of rhythm (*16*–*18*, *22*).

The automaticity of the neural categorization captured here could be suggestive of a low-level process rooted in nonlinearities of the earliest auditory processing stages. However, our modeling results argue against this possibility. Indeed, the neural categories of rhythm identified here could not be explained by nonlinear transformations occurring at the earliest, peripheral stage of the ascending auditory pathway. Rather, our findings highlight a transformation from the representational geometry observed based on modeled auditory nerve responses, mainly tracking the physical temporal structure of the stimuli, toward the categorical representation measured with scalp EEG and consistent with behavioral responses.

Nonetheless, our data do not exclude that rudiments of this transformation could be found already in subcortical auditory nuclei, as has been proposed for the internal representation of periodic beat and meter elicited by rhythmic inputs structured according to an evenly spaced, isochronous grid of time intervals (*59*). In fact, in line with perceptual representations in other domains (*60*), this transformation could be hypothesized to emerge in the form of a gradient of sensitivity from physical to higher-level perceptual features across the processing neural network. For example, a cluster of high-level associative and sensorimotor cortices could support multimodal rhythm representations constrained by contextual and social semantics. These higher-level multimodal representations would thus guide the development and maintenance of categorical representations in sensory regions, for example through dynamic recursive exchanges of inputs (*61*) [i.e., reentry (*62*)]. Along the same line, feedback from higher-level regions may drive categorization in the earliest stages of the auditory pathway via efferent projections (*63*, *64*), which were not captured by the purely feedforward auditory nerve model used here.

Our results are compatible with the view of characteristic “warping” of the representational space where two rhythmic inputs are rendered more similar when they activate internal representation of the same category, as compared to physically equidistant rhythms internally assigned into different categories (*65*). The results of the current study underscore the ability of the fRSA approach to locate boundaries delineating categories in this representational space, thus providing insights into their location and extent. Here, and in line with a large body of prior behavioral work, we identified two rhythm categories: a smaller category encompassing rhythms including the 1:1 ratio and a larger category including the 2:1 ratio and spanning a broad range (between 1.3:1 and 2:1). Critically, the fact that we did not observe a boundary symmetrically splitting the representational space in two equal halves rules out the possibility that the two rhythm categories were driven by nonspecific range effects (*66*).

A potential candidate to account for the observed rhythm categorization could be adaptation. While the absence of a categorical structure in the simulated auditory nerve responses argues against the role of fast low-level adaptation, a slower adaptation produced at later stages of the auditory pathway could, in principle, contribute to the neural categorization observed in scalp EEG activity (*59*, *67*, *68*). Yet, the sharp dissimilarity in responses from each side of the category boundary as identified here would have to be driven by a highly nonlinear adaptation function that would abruptly begin to suppress the response to the tone starting the following rhythmic pattern when the corresponding IOI changes from roughly 290 to 260 ms (i.e., the shortest IOI duration found in the two conditions delimiting the category boundary). However, such an adaptation mechanism has not been described yet in the literature (*69*–*71*) and seems a priori too specific for a deterministic adaptation mechanism.

Instead, the observed categorical boundary might be compatible with a categorization process driven by detection of dissimilarity between the two intervals composing the rhythm (*72*). At first glance, this may seem in agreement with previous behavioral research where two overarching categories of time intervals, even and uneven ones, have been consistently found to span the space of two-interval rhythms (*14*, *19*, *22*, *73*). Accordingly, anisochrony detection, i.e., detection of deviance from evenness, might be one of the relevant mechanisms shaping the neural categorization of rhythm. However, such a process of detection of deviance from isochrony would render the “uneven” category largely uniform over the possible two-interval rhythms, which is hardly compatible with the converging evidence for an underlying prototype or perceptual attractor located near 2:1 ratio (and possible other uneven attractors such as 4:3 and 3:1) (*16*–*19*, *22*, *57*). Indeed, the neural responses recorded in the current study seemed to be systematically biased toward specific temporal templates also found in the behavior and in prior studies (*16*–*19*, *21*, *57*). This result points toward a model where rhythm categories are encoded in prototypical patterns of neural activity (*74*), rather than nonspecific adaptation or anisochrony detection.

All in all, delineating the specific representations underlying the rhythm categories identified in the current study will thus likely require going beyond electrical field potentials recorded with scalp EEG. For example, future work recording single neuron responses in the human temporal (as well as parietal and frontal) cortices appears a promising avenue to progress in our understanding of the neural processes supporting rhythm categorization (*75*–*77*). The fRSA approach proposed here could constitute a major asset in this endeavor, by providing methodological tools to probe these neural responses across a diversity of signals.

Categorical representational geometry has often been closely associated with the concept of a prototype (*25*, *65*). In a Bayesian framework, prototypes can be thought of as modes in the prior distribution of rhythmic stimuli experienced over a lifetime (*27*, *78*). Through probabilistic inference, these priors are then combined with information from sensory organs to support rhythm categorization. In other words, the brain keeps track of what rhythms are encountered in the environment, and the representation of new rhythmic inputs is biased toward the rhythms that are more likely a priori (*22*, *27*). While the prior distribution could be largely unconstrainted (*28*), there are several models that predict intrinsic attraction of rhythm perception toward mathematically simplest small integer ratios (*19*, *26*). For example, influential neurodynamic models of oscillatory networks propose that these biases emerge from predetermined physiological properties of the brain and thus independently of perceiver’s prior experience (*79*).

Indeed, there is abundant behavioral evidence that perception of two-interval rhythms, such as the ones used in the current study, is pulled toward either prototypical 1:1 or 2:1 interval ratios (*16*–*19*, *57*). Yet, previous tapping and perturbation detection studies in humans [see (*16*) for a review], as well as cross-species comparison research based on analysis of vocal communication recordings (*54*, *55*), indicate that the 2:1 prototype may be, in fact, slightly shifted away from the smallest integer ratio. While most studies reported a shift in the direction of 1:1 ratio (i.e., “softening” of the contrast in duration between the two time intervals) (*17*, *18*), others have found shift in the opposite direction (i.e., “sharpening” of the contrast in duration) (*57*).

Our exploratory analysis of the similarity between continuous tapping responses and a set of prototypical rhythmic templates was broadly in line with a shift from exact integer ratios. Within the smaller category, the tapping dynamics mostly resembled a rhythm prototype with a ratio of 0.53, while the larger category featured tapping dynamics highly similar to both a softened (~0.63) and a minimally sharpened (~0.68) prototype. It is worth noting that part of this effect may be explained by a pull toward the center of the range of rhythmic ratios presented in the condition continuum tested here. This possibility could be addressed in future work by adapting the rhythmic stimuli such as to locate the putative small–integer ratio prototypes in the center rather than edges of the condition continuum.

Notably, the tendency to converge toward a limited number of rhythm prototypes was also observed in the EEG responses. That is, there was a general alignment between the prototypes identified in tapping and in the neural responses, with observed prototypes compatible with bias away from mathematically simplest integer ratios. This result thus argues against a critical role of kinematic constraints in driving these more complex ratio prototypes. At the same time, EEG responses featured additional prototypes that were not encountered in the tapping responses. These prototypes included other complex ratios (0.58; approximately corresponding to 4:3) but also small integer ratios such as 0.50 (1:1) and 0.75 (3:1), the latter being in line with prior work probing the internal representation of rhythm indirectly via transient EEG responses elicited by expectation violations (*80*).

Taken together, the heightened similarity to multiple rhythm prototypes per category observed in neural and behavioral data could reflect fingerprints of distinct underlying mechanisms [including individual differences (*16*)]. The interplay between these mechanisms could further shape the rhythmic template eventually selected for perception and overt behavior during an explicit task. Some of these mechanisms might plausibly reflect a privileged role of mathematically simple rhythms emerging from generic oscillatory properties of neural assemblies (*26*, *81*). Others could reflect plastic changes in the neural network acquired through experience [possibly via Hebbian learning (*82*) and in line with recent Bayesian conceptualizations of rhythm perception (*22*, *27*, *28*)].

Building on recent advances in systems neuroscience (*38*), a key advantage of the fRSA approach presented here lies in its capacity to capture categorization without taking potentially limiting or distorting assumptions about the exact form of the response that should reflect categorical information. In other words, the approach only relies on the fundamental definition of the categorization function (*3*–*5*), which requires the system to generate selective (i.e., discriminant) responses to different categories and reproducible (i.e., generalizable) responses when presented with the same category across a wide range of sensory conditions (*1*–*5*).

Compared to previous findings, the fRSA approach goes a critical step beyond methods relying on identification of time intervals between discrete events, which have proven difficult to apply beyond a small set of highly specific responses such as finger tapping (*35*, *40*). Indeed, physiological responses (including brain activity), but also ecologically valid movement elicited by rhythmic sounds [e.g., gestures or dance (*83*, *84*)], typically constitute complex trajectories including smooth fluctuations rather than a series of transient events with clear temporal markers such as onsets (*37*, *83*, *85*, *86*). Therefore, we propose fRSA as a general method that could be applied to any continuous response elicited by periodic rhythmic stimulation and that is, in principle, sensitive to any response feature reflecting categorization as defined in the current study (i.e., generalization and discrimination).

Accordingly, the fRSA approach constitutes an important methodological advance, as it allows rhythm categorization to be probed directly from the dynamics of a wide range of neural responses (e.g., spiking rates, field potentials, oscillatory power fluctuations) at tempi that are ecologically valid [sometimes remarkably fast (*87*)], without relying on overt behavior, thus minimizing decisional, motivational, or motor confounds (*16*, *88*, *89*). By moving beyond behavioral measures, this approach enables testing rhythm categorization in populations where recording a behavioral response is challenging, if not unfeasible (e.g., in preverbal infants). Moreover, as compared to other EEG approaches relying on transient responses elicited by expectation violations (*80*), the fRSA approach allows rhythm categorization to be captured (i) directly (i.e., without relying on additional processes and assumptions), (ii) by explicitly characterizing the representational geometry via dense sampling of the stimulus space (which is precluded by the long testing times typically required to assess EEG responses to deviant events), and (iii) without the need to estimate latency of the neural response (*90*) (which is potentially ill posed, especially at fast, ecologically valid tempi).

This approach thus appears particularly well suited to address long-standing questions about the primitives and roots of musical rhythm, particularly the relative contribution of universal neurobiological constraints shared across species and culture-driven plasticity developing over the course of life through social learning. For example, it could allow us to track how neural rhythm categories develop over the lifespan from birth, how they are shaped by cultural experience or body movement, and how this plasticity is supported by a network of brain regions shared in part by nonhuman species. Therefore, the framework developed in the current study appears promising to bridge the gap between recently found universality of some rhythmic structures in music on the one hand and the vast interindividual and cross-cultural diversity specific to human rhythm perception and production on the other hand.

## MATERIALS AND METHODS

### Participants

Eighteen participants (mean age ± SD = 26.0 ± 4.8 years, 13 females) were recruited in Brussels, Belgium. They reported various levels of musical and dance training (musical training: mean ± SD = 3.7 ± 6.2 years, range: 0 to 21 years, 11 participants never had any musical training; dance training: mean ± SD = 2.7 ± 3.9 years, range: 0 to 12 years, 9 participants never had any dance training). Given prior evidence that rhythm categorization is affected by enculturation rather than the mere amount of practice (*10*, *22*), a mixture of trained and untrained participants was recruited to allow better generalization of our results to the general population. Sample size was estimated in accordance with previous studies using frequency tagging to investigate rhythm perception (*36*, *37*, *49*, *91*). All participants reported to have normal hearing and no history of neurological disorder. The experimental procedure was approved by the local ethical committee of the Saint-Luc Hospital–University of Louvain (project 2018-353) and conducted in accordance with the Declaration of Helsinki. Participants provided written informed consent to participate in the study (with 30 € of financial compensation).

### Stimuli

The stimuli consisted of two-interval rhythmic patterns generated using MATLAB R2022a (MathWorks). The two-interval rhythmic pattern was produced by presenting three auditory events (here, three identical tones) over time, while keeping the total duration of the pattern constant. In such a two-interval pattern, the first interval thus corresponds to the time between the onset of the first and the second event (IOI1, i.e., first IOI), while the second interval (IOI2) is defined as the time between the second and the third event. If the pattern is seamlessly looped, then the third event of one pattern also constitutes the first event of the subsequent pattern (Fig. 1A). Note that this is different from an alternative approach that presented the patterns with interruption or a break [experiment 1 in (*15*, *92*)].

The durations of the two intervals composing repeated two-interval patterns can be expressed as a ratio. For instance, if a given two-interval pattern exhibits a first interval that is twice as long as the second one, we can refer to that pattern as a 2:1 rhythm (Fig. 1A). In the current study, the position of the second tone was varied to obtain 13 interval ratios equally spaced between 1:1 (which can also be expressed as the ratio between the first interval and the total duration of the pattern, i.e., 0.50) and 2:1 (i.e., 0.67). This thus yielded a 13-condition continuum, i.e., with a ratio granularity of 13 increments (Fig. 1B and table S1). We specifically focused on this 1:1-to-2:1 portion of the two-interval space, as this section has been shown to yield the strongest categorical distortion in previous studies (*22*), thus optimizing the likelihood of capturing underlying neural correlates.

The 13 two-interval rhythmic patterns were generated using an identical pure tone of 50-ms duration with a carrier frequency of 300 Hz and a 10-ms linear onset/offset ramp. The patterns had a fixed total duration of 750 ms. This pattern duration was chosen since, on the one hand, it rendered unimanual tapping along with the stimuli reasonably comfortable for adults without musical training (*52*), thus allowing comparisons of our results with the extensive previous work using unimanual tapping tasks (*16*, *18*, *19*, *21*). One the other hand, the chosen pattern duration was expected to yield stronger categorical distortions as opposed to slower tempi, as reported in previous behavioral studies (*16*). Finally, for each of the 13 conditions, the two-interval pattern was then seamlessly looped 30 times to form a 22.5-s-long stimulus sequence (audio S3).

### Experimental procedure

The experiment consisted of six listening blocks and three tapping blocks, with the two types of blocks presented in alternation (a tapping block after every two listening blocks). In all blocks, the 13 different stimulus sequences were presented once in a randomized order.

During the listening blocks, participants were instructed to avoid any unnecessary movement and muscular tension and fixate a cross displayed in front of them to minimize the presence of muscular and ocular artifacts in the EEG recording. Moreover, to ensure attention to the stimulus sequences, we used a task orthogonal to rhythm categorization whereby participants were required to detect transient volume drops in the sequences. The volume drops were obtained by decreasing the amplitude of four consecutive rhythmic patterns within a stimulus sequence to 85% of their amplitude. For each stimulus sequence, there could be one volume drop (occurring in two of the six presentations over all listening blocks), two volume drops (one of six), or none (three of six). After listening to the stimulus sequence without moving, participants verbally reported the number of detected volume drops and received immediate feedback.

During the tapping blocks, participants were instructed to tap in synchrony with the tones using the index finger of their preferred hand. Tapping was performed on a custom-made analog device (hereafter referred to as the “tapping box”) that was positioned by the participants’ side. Participants were instructed not to tap before the beginning of the stimulus sequences to obtain a valid period of baseline before trial onset (stimulus sequences were repeated when not meeting this criterion). Participants were also required not to wait too long to start tapping after the beginning of each stimulus sequence.

The experiment was implemented in MATLAB R2016b (MathWorks, Natick, MA) using the Psychophysics Toolbox extensions (*93*–*95*). The stimulus sequences were presented binaurally through insert earphones (ER-2, Etymotic Research, Elk Grove Village, IL; air-conducted sound from the level of the participant’s clavicle to decrease magnetic interferences) connected to a Fireface UC audio interface [RME Audio, Haimhausen, Germany; sampling frequency 44100 Hz; 74.4-dB SPL (sound pressure level)]. Participants performed the experiment while comfortably seated in a chair with their head resting on the chair support. The experiment had an overall duration of ~90 min including optional breaks between blocks.

### EEG recordings

We recorded brain activity using a 64-channel BioSemi Active Two EEG system (BioSemi, Amsterdam, Netherlands) with two additional channels placed on the left and right mastoids. Recording sites included standard 10-20 system locations (channel coordinates can be found at www.biosemi.com/headcap.htm). Channel offset relative to the common mode sense (CMS) and driven leg (DRL) channel loop was kept below ±50 mV (except in one participant whose offset was kept below ±60 mV but with no impact on the obtained signal-to-noise ratio of the expected responses).

An accelerometer was placed on the head of the participants to monitor whether participants complied with the instructions and avoided head movement during the listening blocks. The signals from all the channels and the accelerometer were digitized at a sample rate of 1024 Hz.

### Behavioral recordings

Tapping responses measured as tapping onsets and continuous force signal were recorded using the tapping box connected to the BioSemi Active Two EEG system’s Analog Input Box. The surface of the tapping box was made of a conductive hard material, thus providing clear tactile feedback. While tapping also produced a small amount of auditory feedback, this was substantially attenuated by the ear inserts used to deliver the auditory stimuli (see above). The device recorded tapping onsets as moments in which the finger got in contact with the conductive surface and closed an electrical circuit. Simultaneously, the force exerted by the finger was recorded as a continuous signal using a six-axis force sensor (FT48224, ATI Industrial Automation, NC). The latency and jitter of the captured signals were below 1 ms, as measured with an oscilloscope.

The tapping onsets were digitized as triggers, while the force signal was digitized as the continuous signal coming from six different sensors of the tapping box at a sampling rate of 1024 Hz. In addition, we also recorded a copy of the delivered acoustic signal through the BioSemi Active Two EEG system’s Analog Input Box to control for latency in the recording system, which was digitized at 1024 Hz.

### Auditory nerve modeling

To simulate responses elicited by the rhythmic stimuli in a set of auditory nerve fibers, we used an auditory nerve model developed by Bruce *et al.* (*51*) as implemented in UR_EAR toolbox (version 2020b). Specifically, we modeled responses from 128 cochlear channels with characteristic frequencies logarithmically spaced between 130 and 3000 Hz. The default parameters used for cochlear tuning matched data available from human participants (*96*). For each channel, we simulated a biologically plausible distribution of high–, mid–, and low–spontaneous rate fibers (8, 12, and 31, respectively, i.e., 51 fibers in total) (*97*). The resulting instantaneous firing rate was then summed across fibers and cochlear channels (*98*, *99*), yielding an estimate of the variation in firing rate over the course of the 22.5-s-long stimulus sequence in each condition.

### Statistical analysis

EEG and behavioral data were analyzed using Letswave 6 (https://github.com/NOCIONS/letswave6), Letswave 7 (https://github.com/NOCIONS/letswave7), and custom-built scripts running on MATLAB R2022a (MathWorks).

### EEG data preprocessing

A Butterworth high-pass filter (fourth order, cutoff at 0.1 Hz) and low-pass filter (fourth order, cutoff at 64 Hz) were applied to raw continuous EEG data to remove slow drifts and responses at very high frequencies irrelevant to the current study. We subsequently downsampled the data to 256 Hz (i.e., by a factor of 4) to facilitate data handling and storage. We segmented the continuous data from −5 s to 27.5 s with respect to the onset of each stimulus sequence before performing artifact rejection. Following visual inspection of the data, we linearly interpolated noisy channels with the three closest neighboring channels (two channels in 1 participant, one channel in 2 participants, and no channels in the remaining 15 participants).

We then applied independent component analysis (ICA) to remove artifactual components due to blinks and eye movements. ICA matrices were computed from data preprocessed the same way as described above, except that we used a higher high-pass filter cutoff (1 Hz; fourth-order Butterworth filter) to improve artifact classification accuracy (*100*). The data were resegmented from 0 to 22.5 s with respect to the onset of the stimulus sequence (i.e., corresponding to each trial duration), and ICA matrices were obtained using the probabilistic ICA model with Laplace approximation. Artifactual independent components (ICs) due to blinks and eye movements were identified from visual inspection of the ICs time courses and topographies and then removed (two ICs removed in 3 participants, one IC removed in 14 participants, and no IC removed in 1 participant). After this step, the preprocessing pipeline applied to the EEG data diverged depending on whether the data were then further analyzed in the frequency or in the time domain.

### EEG frequency domain analysis

After artifact rejection, the data were resegmented from 0 to 22.5 s (i.e., total duration of individual stimulus sequences) relative to stimulus sequence onset. The duration of the resulting epochs thus corresponded to an exact integer multiple of the rhythmic pattern duration, hence preventing spectral leakage of responses at the frequencies of interest (determined as 1/pattern duration and harmonics) into the surrounding frequency bins after applying the Fourier transform (*101*).

The data were re-referenced to average mastoids with the goal of maximizing the EEG responses to the acoustic stimuli (*37*, *45*). The obtained epochs were then averaged in the time domain separately for each channel, condition, and participant to attenuate EEG activity not phase-locked with the stimulation. We then applied a fast Fourier transform (FFT) yielding complex spectra for each channel, participant, and condition with 0.044-Hz frequency resolution, corresponding to the difference in frequency between two consecutive frequency bins (i.e., 1/22.5-s stimulus sequence duration).

To extract relevant features characterizing the neural response in each condition, we capitalized on the fact that the spectrum of any signal that is systematically repeated with a fixed repetition rate (i.e., periodically) will only contain peaks at specific frequencies corresponding the repetition rate (i.e., *f* = 1/repetition period) and its integer multiples (i.e., harmonics, 2*f*, 3*f*, 4*f*, etc.) (*34*, *41*, *101*, *102*). In the current study, the stimulus sequences were composed of a seamlessly repeated rhythmic pattern presented with an exact fixed repetition period (i.e., 750 ms). Therefore, the spectrum of any response systematically elicited across repetitions of the rhythm pattern is expected to contain responses at frequencies corresponding to $f=\frac{1}{\text{pattern duration}}$ and harmonics (i.e., *f* = 1/0.75 s = 1.33 Hz, 2*f* = 2.66 Hz, …), which were thus considered as frequencies of interest in further analyses.

Following a procedure adopted in previous frequency-tagging studies (*42*–*44*), we first assessed the significance of the response at frequencies of interest at the group level. To do so, we computed the magnitude spectrum (i.e., absolute value of the complex spectrum) separately for each participant, condition, and channel. We then computed the grand average across participants, conditions, and all channels excluding the mastoids (i.e., 64 channels). *z*-scores at the frequencies of interests were calculated as the difference between the magnitude at the frequency bin of interest and the averaged magnitude of eight surrounding bins (four on each side, excluding the immediately adjacent bins to avoid potential remaining spectral leakage), divided by the standard deviation (SD) of the same selected bins (*z*-score $=\frac{x-\mathrm{bl}}{\text{SD}\left(\mathrm{bl}\right)}$ ). The response at a given frequency was considered significant if the corresponding *z*-score was higher than 3.09 (i.e., *P* < 0.001, one-tailed, signal > noise). Consecutive significant frequencies of interest were considered for the following analysis (see fig. S3).

### EEG time-domain analysis

After artifact rejection, we applied a fourth-order Butterworth low-pass filter with a 10-Hz cutoff (i.e., to match the frequency range showing significant consecutive harmonics in the obtained EEG spectra, as measured using the *z*-score procedure described above and as further used in fRSA analyses; see fig. S3). The data were segmented from the onset to the end of the stimulus sequence (0 to 22.5 s) and re-referenced to average mastoids. The data were then further segmented to obtain successive chunks corresponding to the rhythmic pattern duration (i.e., 750 ms) and downsampled by a factor of 4 (i.e., to a 64-Hz sampling rate) to reduce dimensionality. This yielded a total of 180 epochs (6 trials × 30 pattern repetitions) per condition, channel, and participant. To correct for possible offsets, the data was demeaned by subtracting at each time point within each epoch the average amplitude measured over the epoch for a given channel. The resulting epochs were then averaged separately for each condition, channel, and participant.

### Behavioral data preprocessing—ITIs

To analyze ITIs, we adopted a procedure followed in previous sensorimotor synchronization studies (*18*, *22*). First, we cleaned the series of tap onsets recorded as exact time stamps when the finger got in contact with the tapping box. The cleaning procedure was necessary since computing the ITI ratio for each repetition of the rhythmic pattern requires exactly three tap onsets. Accidental extra taps were removed by discarding tap onsets that were not separated from the preceding tap by at least 30 ms. Moreover, to discard responses that reflected attentional lapses or motor errors, we carried out the following steps. First, we corrected for the fact that humans tend to tap slightly before the pacing stimulus (*52*). For each participant, we matched each tap onset with the closest tone and calculated the mean tap-tone asynchrony across all trials and conditions. We then subtracted the mean asynchrony from the onset time of each tap. All taps where the absolute value of the residual asynchrony to the closest tone onset was greater than 80 ms (i.e., less than half of the smallest IOI in the stimulus, which corresponds to the second interval in the 2:1 condition, 250 ms) were excluded from further analysis.

The obtained tap onsets were then used to calculate the ITIs separately for each repetition of the rhythmic pattern. Each tone in the given repetition was matched with the closest mean asynchrony–corrected tap. Then, we measured the time interval between the first and the second tap (ITI1) and the time interval between the second tap and the tap paired with the first tone of the directly following rhythm repetition (ITI2). The ITI ratio was calculated as ${\text{ITI}}_{\text{ratio}}=\frac{\text{ITI}1}{\text{ITI}1+\text{ITI}2}$ . In case any of the tones was not matched with a tap, this precluded the computation of the ITI ratio, and the whole rhythmic pattern repetition was excluded from further analysis. The computed ITI ratios were then averaged across pattern repetitions separately for each participant and condition.

### Behavioral data preprocessing—Tapping onset time series

Separately for each participant, condition, and tapping trial, we created a continuous time-domain signal with duration corresponding to the length of the stimulus sequence and 256-Hz sampling rate. The value of each sample corresponding to a tap-onset time was set to 1 (i.e., a unit impulse) and 0 otherwise. Note that all tap onsets detected by the tapping box were used without any further preprocessing (i.e., unlike for the ITI analysis above).

### Behavioral data preprocessing—Continuous tapping force

For each participant, condition, and tapping trial, the continuous tapping force recorded from the six force sensors of the tapping box was segmented from −1 to 22.5 s with respect to onset of the stimulus sequences. For each sensor, the force signal recorded over the trial duration was baseline corrected by subtracting at each time point the averaged signal over 1 s before trial onset to correct for potential offsets present in the recordings. The signal from the six sensors was then combined (using the device calibration matrix) to obtain the continuous tapping force orthogonal to the tapping box. The obtained tap-force signals were resegmented from 0 to 22.5 s (i.e., stimulus sequence duration) relative to the onset of the stimulus sequences and downsampled to 256 Hz.

### Behavioral data frequency domain analysis

The continuous responses (both continuous tap-onset time series and tapping force signals) were averaged across trials corresponding to different repetitions of the same condition, and an FFT was applied to obtain a response spectrum for each condition and participant. We assessed the significance of the responses at frequencies of interest at the group level (see fig. S3). These computations were performed on the magnitude spectrum averaged across all participants and conditions, following the same steps as for the EEG responses.

### Behavioral data time-domain analysis—ITIs

The average ITI ratios were collapsed across participants and fitted either with a linear or a sigmoid model. Parameters were estimated by minimizing the least-squares error, and the performance of each model was evaluated using leave-one-participant-out cross-validation. The sigmoid model was also fitted separately for each individual participant.

To test whether the tapped interval ratios overall significantly deviated from the stimulus ratios, we divided the range of stimulus ratios into 13 equal bins, computed histogram of the number of ITI ratios in each bin, and compared it to the one obtained under null hypothesis of a uniform distribution using the χ^2^ goodness of fit statistic.

### Behavioral data time-domain analysis—Continuous tapping force

The continuous tapping force signals were also analyzed in the time domain, similarly to the EEG responses. To do so, a fourth-order Butterworth low-pass filter with an 18-Hz cutoff was first applied to the preprocessed continuous tapping force signals (i.e., matching the frequency range whereby significant responses were identified using the procedure described above; see fig. S3). Similarly to the EEG analysis, the data were then segmented into successive chunks of 750-ms duration, starting from onset time until the end of the stimulus sequence, thus yielding 90 epochs (3 trials × 30 pattern repetitions) per condition and participant, downsampled by a factor of 4, demeaned, and averaged separately for each condition and participant.

### Representational similarity analyses

Neural and behavioral data were analyzed in the RSA framework (*38*, *39*). RSA is a pattern information analysis comparing representational geometries that can be built from stimulus descriptors, empirical data, and conceptual and computational models. Crucially, by moving from a unit-specific to a unit-free space, RSA allows to investigate whether a brain response reflects stimulus properties, higher-level categories, or aspects of overt behavior. To characterize the representational geometries of the different types of data obtained in the current experiment, we computed several RSMs reflecting pairwise similarities across conditions. These RSMs were all characterized by a diagonal corresponding to the similarity between the signal and itself for each condition, thus, with maximal similarity, separating the RSM into a lower and upper triangular half with strict symmetry between the halves.

The acoustic RSM was obtained by taking the ratio between the first IOI and the total duration of the rhythmic pattern and computing the absolute difference of this value across all pairs of conditions. The resulting difference values were subtracted from 1 to yield a similarity matrix with ones on the diagonal. This RSM thus reflects the equal spacing of the rhythmic ratios along the condition continuum, i.e., a linear decrease in similarity across conditions (Fig. 1B).

For each participant, a neural RSM was obtained as follows. First, the real and imaginary coefficients of the complex Fourier spectrum at the frequencies of interest (i.e., harmonics of the rhythmic pattern repetition rate where a significant response was observed at the group level) were extracted separately for each of the 64 channels. Then, separately for each condition, these values were concatenated into a feature vector, yielding number of dimensions equal to *n* frequencies of interest × 2 (real and imaginary coefficient) × 64 channels. Finally, we computed the similarity of these feature vectors using Pearson’s correlation across all pairs of conditions. In addition, we evaluated whether considering all 64 EEG channels and thus accounting for any individual differences in response topography is critical to reveal the categorical geometry of neural responses. To this end, we built the neural RSM from complex coefficients averaged across nine frontocentral channels (F1, Fz, F2, FC1, FCz, FC2, C1, Cz, and C2). Note that this is equivalent to extracting the coefficients from complex spectra of the channel-averaged time course. Channel selection was motivated by the observation that responses recorded over these channels seem to consistently capture EEG responses to repeating acoustic rhythms (*36*, *48*–*50*) (see also fig. S3). Finally, a neural RSM was also built from time-domain EEG responses. To do so, the preprocessed epochs corresponding to the average amplitude values measured over the time course of the rhythmic pattern were averaged across nine frontocentral channels (F1, Fz, F2, FC1, FCz, FC2, C1, Cz, and C2) separately for each participant and condition. Then, for each participant, the similarity of these time-domain responses across conditions was calculated using Pearson’s correlation across all pairs of conditions.

We then built behavioral RSMs from the various tapping signals (i.e., from discrete to continuous tapping signals). Individual ITI RSMs were obtained by computing the absolute difference between the produced average ITI ratio across all pairs of conditions. To capture similarity, the difference values were subtracted from 1 as for the acoustic RSM. Moreover, individual tap-onset RSMs were built, as for the neural responses, using feature vectors obtained by concatenating the real and imaginary coefficients at the frequencies of interest from the complex spectrum of the continuous tap-onset signals separately for each condition and participant (number of dimensions = 2 × *n* frequencies). The tap-onset RSM was then obtained for each participant by computing Pearson’s correlations between these feature vectors across all pairs of conditions. Additionally, individual tap-force RSMs were obtained from continuous tapping force signal by following the same procedure as described for the tap-onset RSMs above. Finally, as for the neural responses, individual tap-force RSMs were also built by comparing the time-domain force values averaged across repetitions of the rhythmic pattern across all pairs of conditions using Pearson’s correlation.

Further, we computed an RSM with the responses obtained from the auditory nerve model. As for the neural responses, the complex spectrum of the auditory nerve model responses was calculated for each condition using FFT. To obtain an RSM directly comparable with the neural RSM, we extracted real and imaginary coefficients at the same frequencies of interest as for the EEG responses and concatenated them into a feature vector separately for each condition. The auditory nerve RSM was obtained by calculating Pearson’s correlation between feature vectors across all pairs of conditions.

### Theoretical models of rhythm categorization

Theoretical models of rhythm categorization were built based on the fundamental definition of a categorization function: maximal similarity of responses across conditions within the same category (thus setting pairwise similarity across these conditions to 1) and maximal dissimilarity of responses across different categories (thus setting the pairwise similarity of the corresponding conditions to 0).

Given that the stimuli used in the current study are expected to elicit the perception of two rhythm categories (*10*, *14*, *15*, *22*), we built a set of 10 two-category models. The models differed from each other in terms of the position of the category boundary, i.e., in terms of the experimental condition where the switch from one category to the other would occur (Fig. 1E). Models where one of the categories spanned less than two conditions were excluded from the analysis, given that they cannot test within-category generalization. Rather than assuming the position of the category boundary a priori, having several theoretical models allowed us to estimate it from the data separately for each participant, thus accounting for any potential individual differences (*16*). Likewise, we were able to assess whether the preferred theoretical models were consistent across participants and across neural and behavioral responses.

### Evaluation of shared structure in RSMs

To investigate whether a response reflected rhythm categorization, we performed partial correlations between individual RSMs and the RSM of each theoretical model of rhythm categorization while partialing out the acoustic RSM to account for any representational structure driven by the stimulus (Fig. 1E). To this end, we calculated Spearman’s partial correlations between the lower triangular parts of the RSMs, excluding values from the diagonal to avoid inflating correlation values (*103*). The best-fitting categorical model was defined as the one with the highest correlation coefficient.

Significance of the result for each individual participant was evaluated using permutation testing (5000 iterations), with the aim to probe whether the partial correlation coefficient with the best-fitting categorical model is higher than what would be expected from chance. In each iteration, we randomly shuffled the values of the lower triangular response RSM and computed the partial correlation between the shuffled response RSM and the theoretical categorical models while partialing out the acoustic RSM. The correlation value corresponding to the winning theoretical categorical model identified from shuffled response RSM was stored for each iteration. These values were used to build a null distribution of correlation values for statistical testing.

Significance was also assessed at the group level (permutation test, 10,000 iterations) to test whether the group-averaged partial correlation coefficient with the participant-wise best-fitting model is significantly higher than expected from chance. For each iteration, we shuffled all individual RSMs and found the best-fitting categorical model for each participant in the same way as described above. The average correlation coefficient of the best-fitting categorical model was stored to build a null distribution.

A *P*-value was calculated as the proportion of observations in the null distribution that had a correlation value higher than the one observed in the measured data (*104*). To correct for multiple comparisons, *P* values were adjusted using the Bonferroni correction for the 10 theoretical categorical models.

Next, we were interested in comparing the RSMs obtained for different kinds of responses, such as ITI RSM, tap-onset RSM, tap-force RSM, and neural RSM. These comparisons were carried out using the following methods.

To test whether two RSMs shared similar structure beyond what could be explained by the stimuli, we calculated Spearman’s partial correlation between the lower triangular parts of both RSMs while including the acoustic RSM as a covariate. Significance of the correlation was tested using a permutation test (5000 iteration), where partial correlation was computed from randomly shuffled RSMs on each iteration to build a null distribution of correlation coefficients. The same permutation procedure was used to establish the significance of the average correlation coefficient across participants, i.e., a group level test (10,000 iterations).

To test how prominent was the observed categorical structure at the group-level between two kinds of responses, the individual Spearman’s correlation coefficients obtained for the best-fitting model were Fisher transformed and further compared across the two kinds of responses using a paired *t* test. In addition, Bayes factors (BF_10_) were calculated quantify the evidence in favor of the alternative hypothesis (H_1_) over the null hypothesis (H_0_), as implemented in the package bayesFactor for MATLAB (https://github.com/klabhub/bayesFactor).

Last, to test whether two kinds of responses showed a similar category boundary position, we took into account the fact that theoretical models with similar category boundary positions were highly correlated. Hence, instead of relying on the best-fitting model, we considered all the possible models. For each response, individual Spearman’s partial correlation coefficients were obtained by correlating the corresponding RSM with each of the 10 theoretical models of categorization. This yielded vectors of 10 correlation coefficients, which were first Fisher transformed and then correlated (Pearson’s correlation) between the two kinds of responses separately for each participant. The obtained correlation values were then Fisher transformed and tested against zero using a one-sample *t* test, to test whether the correlation in the distribution of fit across category boundaries was similar between the two responses at the group level.

### Prototype analysis

To shed light on the properties of the signals making up the categorical structure observed from brain and behavioral responses, we evaluated the similarity of the continuous response (either tapping or EEG) in each condition and a set of prototypical signals.

We built 76 time-domain prototypes with the same duration as the rhythmic sequences (i.e., 22.5 s). Each prototype was made of 30 seamlessly repeating 750-ms rhythmic patterns comprising two impulses arranged over time to create two IOIs with a given ratio. Across prototypes, the IOI ratios were equally spaced between a ratio of 0.50 (i.e., 1:1 ratio) and 0.84 (i.e., with contrast in duration between the two intervals sharper than the 3:1 ratio).

Then, we characterized the temporal structure of each prototype, that is, the profile of (a)symmetries within the span of the repeating 750-ms pattern by computing the spectrum of each prototype using FFT and extracting a vector of magnitudes at the frequencies of interest, as selected for the analyzed responses (i.e., harmonics of the pattern repetition rate up to 8 and 16 Hz for neural and tapping responses, respectively).

Likewise, we used FFT to obtain the magnitude spectrum of the analyzed response separately for each condition and participant. To minimize the contribution of broadband noise to the magnitudes measured at the frequencies of interest, we applied a noise correction procedure by subtracting from each frequency bin of interest the local noise baseline approximated as the average magnitude at eight surrounding frequency bins (four on each side, excluding the immediately adjacent bins to avoid potential remaining spectral leakage).

The similarity between each prototype signal and the response in each condition was evaluated using a bootstrapping procedure (*105*) as follows (see fig. S7). We selected a random sample of 18 participants with replacement (i.e., each of the 18 participants could be selected multiple times or not at all). The magnitude spectra of the selected participants were then averaged, and a vector of magnitude values at the frequencies of interest was obtained for each condition. These grand-average magnitude vectors were correlated with the vectors corresponding to each of the 76 prototypes, and the maximally correlated prototype was stored. This procedure was repeated 1000 times, thus yielding a distribution of maximally correlated prototypes separately for each condition (fig. S7).

To test whether the distribution of maximally correlated prototypes was concentrated near the stimulus ratio in each condition, we tested whether the maximally correlated prototype fell within a narrow window centered on the stimulus ratio. The width of the window was delimited by the midpoints between successive ratios on the condition continuum. That is, the window started halfway between the tested and the directly preceding condition and ended halfway between the tested and the directly following condition. The number of maximally correlated prototypes inside the window was then subtracted from the number falling outside of the window, in each case weighted according to the size of the respective range (i.e., by dividing these numbers by the rhythmic ratio range covered by the “in” and “out” window, respectively) (*54*). A resulting in-out window index larger than zero would indicate that the maximally correlated prototype is likely located close to the stimulus ratio, rather than away from it. To obtain a CI on the in-out window index, we repeated the bootstrap procedure 500 times. If the index was smaller than 0 more than 95% of times, then we considered the most correlated prototype to have a different interval ratio than the one of the stimulus (Bonferroni corrected, i.e., multiplied by the number of conditions).

A similar approach was used to localize peaks in the distribution of maximally correlated prototypes. This was done by first splitting the condition continuum into two segments based on the category boundary identified using fRSA (median category boundary across participants) and collapsing the distributions separately for each of the two obtained subsets of conditions. For each subset, local peaks in the pooled distribution were identified by first calculating a kernel smoothing function estimate using MATLAB’s “ksdensity” function and locating peaks higher than 10% of the average density value using the “findpeaks” function. To test the significance of each located peak, we counted the maximally correlated prototypes within a narrow window centered on the peak (width corresponding to the spacing between neighboring stimuli on the condition continuum) and compared this to the counts falling in regions directly flanking the window (equivalent to half the window width on each side). Statistical significance was evaluated by repeating the bootstrap procedure 500 times and computing a *P* value as a proportion of times the resulting in-out window index was larger than zero (Bonferroni corrected, i.e., multiplied by the total number of tested peaks).

## Supplementary Materials

### The PDF file includes:

Figs. S1 to S7 Tables S1 and S2 Legends for audios S1 to S3

### Other Supplementary Material for this manuscript includes the following:

Audios S1 to S3
