# Supplementary material for: Revealing rhythm categorization in human brain activity
Source: Sci Adv. 2025 Jul 30;11(31):eadu9838. doi: 10.1126/sciadv.adu9838 (PMC12309689; doi:10.1126/sciadv.adu9838)
Supplement: Supplementary file 1 — Figs. S1 to S7 Tables S1 and S2 Legends for audios S1 to S3 [file sciadv.adu9838_sm.pdf]

Science Advances
AAAS

Supplementary Materials for

Revealing rhythm categorization in human brain activity

Francesca M. Barbero *et al.*

Corresponding author: Francesca M. Barbero, francesca.barbero@uclouvain.be; Tomas Lenc, t.lenc@bcbl.eu; Sylvie Nozaradan, sylvie.nozaradan@uclouvain.be

Sci. Adv. **11**, eadu9838 (2025)
DOI: 10.1126/sciadv.adu9838

The PDF file includes:

Figs. S1 to S7
Tables S1 and S2
Legends for audios S1 to S3

Other Supplementary Material for this manuscript includes the following:

Audios S1 to S3

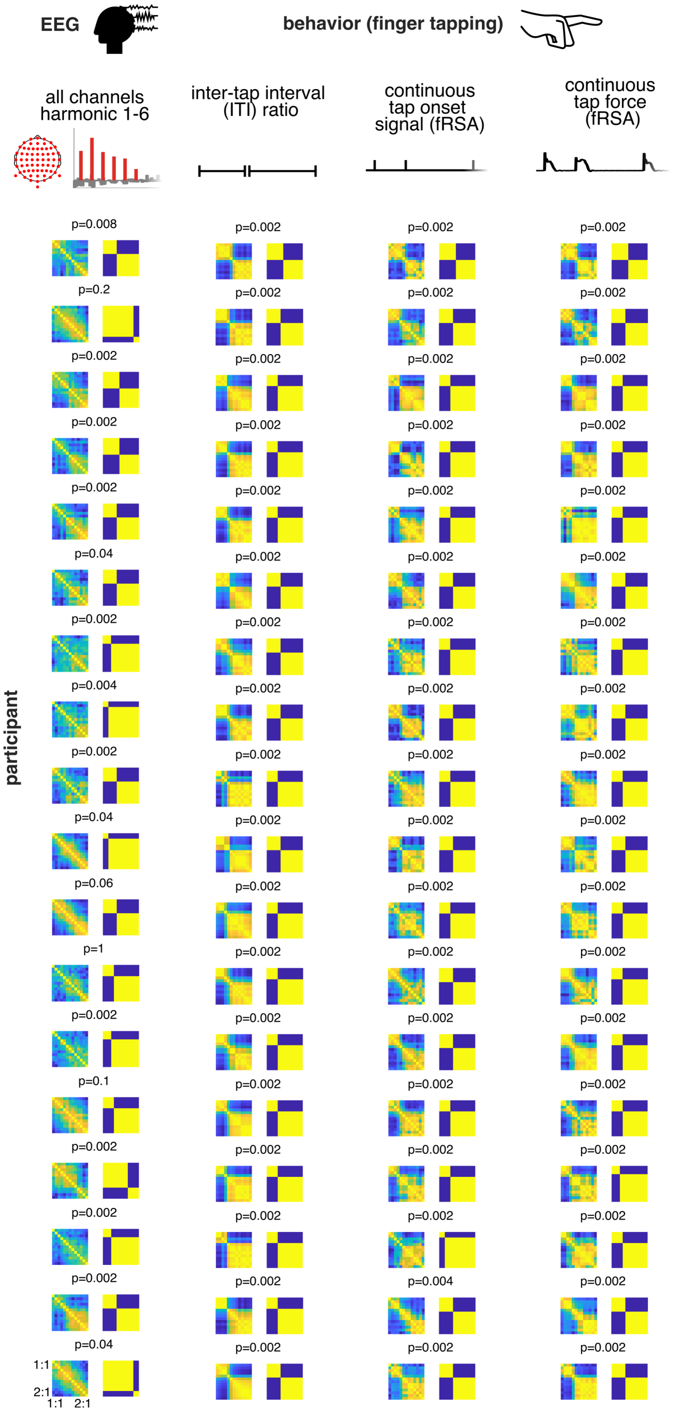

Fig. S1. Representational Similarity Matrices (RSMs) of individual participants show overall consistent categorization and boundary locations. Each row corresponds to a single participant. Neural RSMs were obtained using fRSA by considering frequencies up to 8 Hz and all 64 EEG channels. The RSMs for ITI ratios were obtained from pairwise absolute differences in the produced ITI ratio across conditions. The RSMs for continuous tap onset and force signals were obtained with fRSA considering frequencies up to 16 Hz.

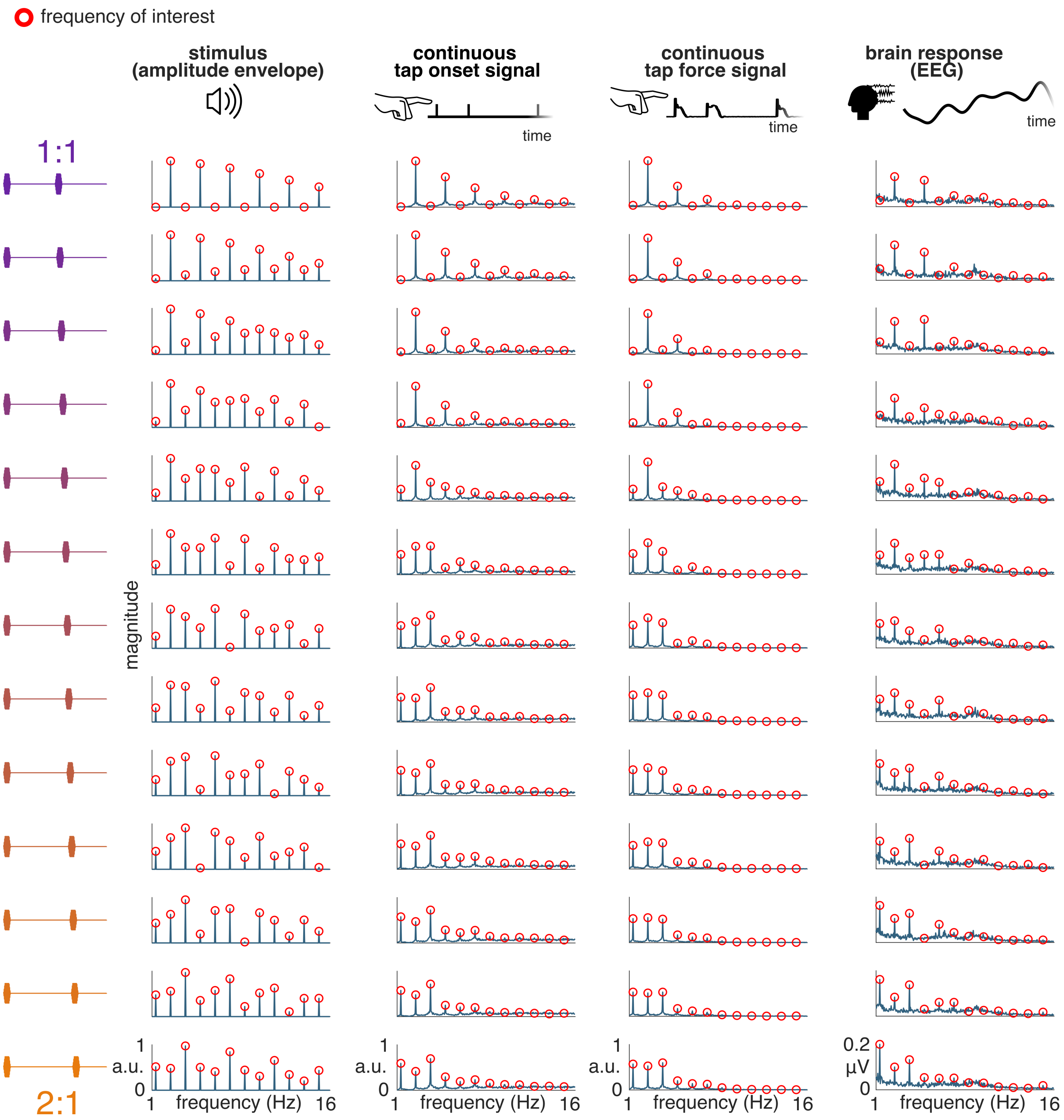

Fig. S2. Grand average magnitude spectra of the stimulus, EEG, and tapping responses show peaks at a priori determined frequencies of interest. Grand average magnitude spectra shown separately for each condition. The stimulus spectrum is computed from the amplitude envelope of the auditory stimulus. Red circles highlight frequencies corresponding to the rate of rhythmic pattern repetition and harmonics.

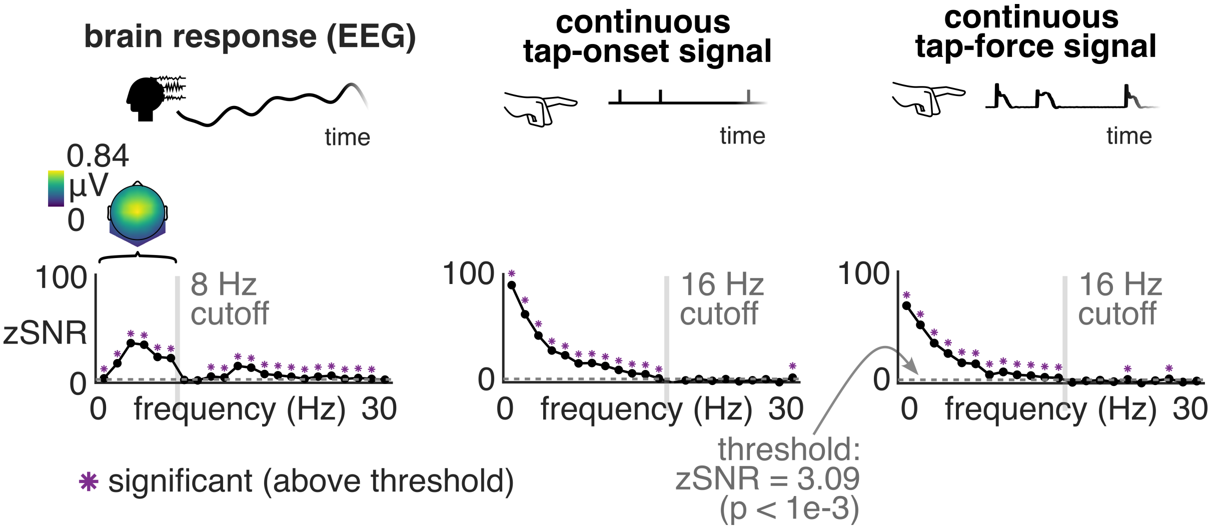

Fig. S3. Significance of response harmonics. Z-scored values of signal-to-noise ratio (zSNR) of the response at each harmonic of the rhythmic pattern repetition rate obtained from the grand-average spectrum of the EEG and continuous tapping responses. The horizontal dashed gray line indicates the threshold corresponding to z-score 3.09 (equivalent to $p < 0.001$, one-tailed test, testing signal > noise). The zSNR value for each harmonic is shown as a black circle, and the magenta asterisk above indicates that the corresponding value is above threshold. A vertical grey line indicates the location of the cutoff used to build the RSMs (set to 8 Hz for neural and 16 Hz for continuous tapping responses). A topographical map of the magnitude summed across first 6 harmonics (i.e. up to 8 Hz, indicated by black brackets) is shown above the EEG plot.

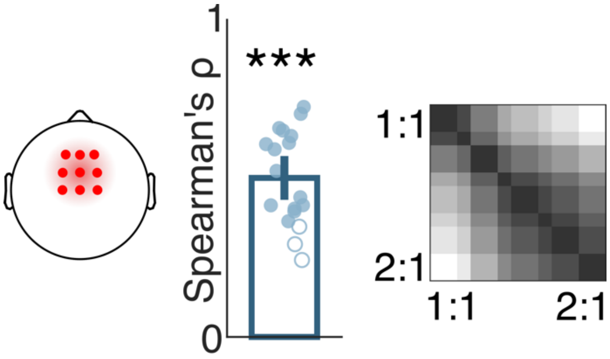

Fig. S4. Similar categorization was obtained when applying fRSA to EEG responses averaged across fronto-central channels. Same as Figure 4, but with fRSA applied to a time course of the EEG response averaged across 9 fronto-central channels and considering frequencies up to 8 Hz. Blue circles show Spearman's correlation of the EEG response RSMs with the best-fitting categorical model, obtained separately for each participant. Filled circles indicate a significant permutation test at the individual participant level, and asterisks indicate a significant permutation test at the group level (Bonferroni corrected, *p < 0.05, **p < 0.01, ***p < 0.001). Overlay of best-fitting significant categorical models across participants is shown on the right.

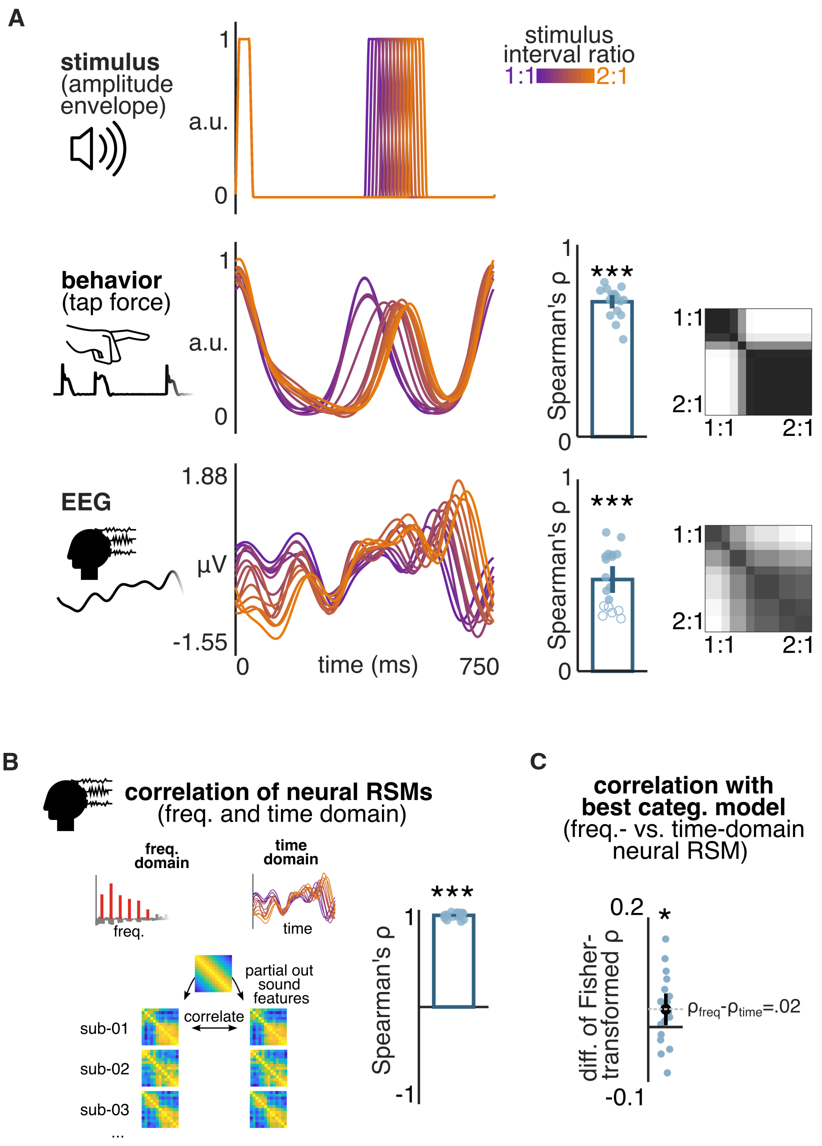

Fig. S5. Similar categorization was obtained with time-domain analysis. (**A**) The panel on the left shows an overlay of the grand-average time-domain response to a single repetition of the rhythmic pattern separately for each condition (indicated by the color gradient). The panel on the right shows (i) the correlation of a categorical model with the time-domain RSM based on the similarity of time-domain responses across conditions, and (ii) an overlay of best-fitting significant categorical models across participants. Filled circles indicate a significant permutation test at the individual participant level, and asterisks indicate a significant permutation test at the group level (Bonferroni corrected, $*p < 0.05$, $**p < 0.01$, $***p < 0.001$). (**B**) Participant-wise correlation of the neural RSM based on the frequency-domain analysis (considering frequencies up to 8 Hz) and the corresponding neural RSM based on the time-domain analysis. Both RSMs were built from the average response across 9 fronto-central channels. The shared information driven by stimulus features was removed by partialling out the acoustic model RSM. Data from individual participants are shown as blue circles (filled circle indicates a significant permutation test at the individual participant level). Error bars represent a 95% confidence interval. Asterisks indicate a significant permutation test at the group level ($***p < 0.001$). (**C**) Difference of Fisher-transformed

correlations with the best categorical model obtained from the frequency- and time-domain analyses of the EEG responses. Blue circles correspond to individual participants, error bars represent a 95% confidence interval, and asterisks indicate a significant t-test against zero (*$p < 0.05$).

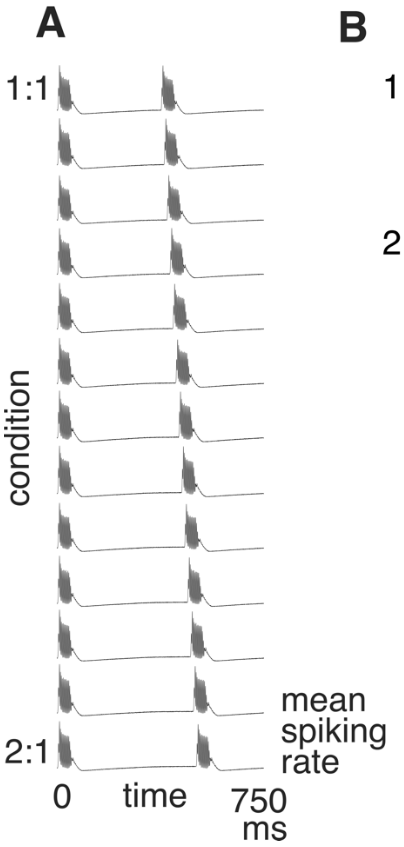

Fig. S6. Auditory nerve model showing an absence of two-category geometry. (**A**) Auditory nerve response to an average repetition of the 750-ms long rhythmic pattern separately for each condition. (**B**) Auditory-nerve RSM built using fRSA and considering frequencies up to 8 Hz (i.e., same as for the EEG analysis).

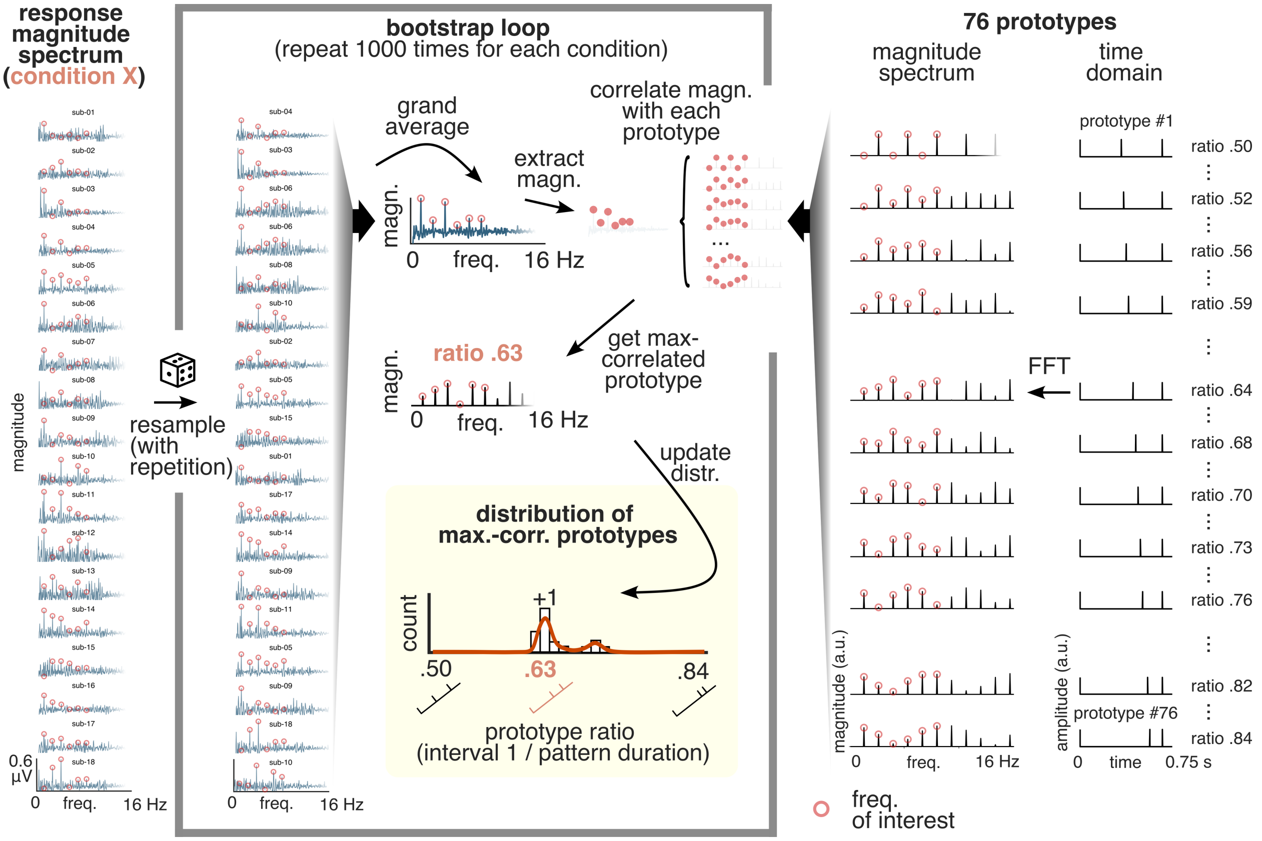

Fig. S7. Similarity with two-interval prototypes was evaluated using magnitude spectra and bootstrapping. Schematic of the analysis to evaluate similarity between the response and a set of prototypical template signals. The prototypes were made by concatenating series of identical unit impulses, arranged to create a repeating 750-ms long pattern of two intervals with ratios equally spaced between 0.50 and 0.84, thus yielding 76 prototypical template signals in total. The time-domain representation of the constituent pattern is shown on the right for several example prototypes. The magnitude spectra of these example prototypes are depicted next to their time-domain representation, with frequencies of interest (i.e. harmonics of the repeating pattern rate) highlighted with red circles. Individual EEG magnitude spectra from an example condition are shown on the left. The grey box in the middle highlights one iteration of the bootstrapping procedure. The vector of magnitudes at the frequencies of interest (red circles) is extracted from the grand-average spectrum obtained by re-sampling, and correlated with the corresponding vector of each prototype, from which the maximally correlated prototype is selected. Repeating this procedure 1000 times yields a distribution of maximally correlated prototype for each condition.

condition	IOI 1 (s)	IOI 2 (s)	IOI 1 ratio	IOI 2 ratio
1 (1:1)	0.375	0.375	0.5	0.5
2	0.386	0.364	0.514	0.486
3	0.396	0.354	0.528	0.472
4	0.407	0.343	0.543	0.458
5	0.418	0.333	0.557	0.443
6	0.428	0.322	0.571	0.429
7	0.439	0.311	0.585	0.415
8	0.449	0.301	0.599	0.401
9	0.46	0.29	0.613	0.387
10	0.471	0.279	0.628	0.373
11	0.481	0.269	0.642	0.358
12	0.492	0.258	0.656	0.344
13 (2:1)	0.503	0.248	0.67	0.33

Table S1. Inter-onset intervals (IOIs) used for stimulus construction. The table shows the interval (in seconds) between the onset of the first and the second tone (IOI 1), and between the second tone and the first tone of the following repetition of the rhythmic pattern (IOI 2). The table also shows the ratio between each IOI and the total duration of the rhythmic pattern. The values are shown separately for each of the 13 conditions, equally spaced between ratio 1:1 (equivalent to IOI 1 ratio 0.50) and 2:1 (equivalent to IOI 2 ratio 0.67).

	EEG				tap force			
participant	zSNR	boundary	rho	p_{bonf}	zSNR	boundary	rho	p_{bonf}
1	2.35	0.564	0.44	0.0079984	13.73	0.578	0.73	0.0019996
2	3.52	0.649	0.32	0.17796441	10.86	0.564	0.62	0.0019996
3	2.6	0.578	0.64	0.0019996	16.88	0.55	0.82	0.0019996
4	3.28	0.578	0.58	0.0019996	28.99	0.55	0.77	0.0019996
5	4.32	0.564	0.64	0.0019996	21.73	0.55	0.78	0.0019996
6	2.98	0.564	0.38	0.0379924	13.97	0.564	0.77	0.0019996
7	1.54	0.535	0.51	0.0019996	16.42	0.55	0.6	0.0019996
8	1.9	0.521	0.48	0.0039992	15.51	0.564	0.67	0.0019996
9	2.78	0.564	0.61	0.0019996	10.24	0.55	0.75	0.0019996
10	5.75	0.521	0.37	0.0439912	20.09	0.564	0.74	0.0019996
11	7.47	0.564	0.36	0.0619876	14.09	0.55	0.72	0.0019996
12	1.28	0.55	0.23	1	7.23	0.55	0.64	0.0019996
13	2.05	0.535	0.52	0.0019996	14.09	0.55	0.73	0.0019996
14	6.27	0.55	0.35	0.1139772	12.93	0.55	0.69	0.0019996
15	2.75	0.62	0.75	0.0019996	10.07	0.535	0.73	0.0019996
16	2.3	0.535	0.54	0.0019996	11.88	0.55	0.72	0.0019996
17	2.58	0.564	0.72	0.0019996	17.04	0.564	0.49	0.0019996
18	4.76	0.649	0.38	0.0359928	19.54	0.564	0.74	0.0019996

Table S2. Summary of fRSA results separately for each individual participant. The results obtained with fRSA are shown separately for each participant and for the neural response (considering frequencies up to 8 Hz and 64 EEG channels) and for the tap-force response (considering frequencies up to 16 Hz). For each type of response, the table shows the (i) z-scored values of signal-to-noise ratio (zSNR), (ii) rhythmic ratio corresponding to the boundary of the best-fitting categorical model, (iii) Spearman's correlation of the best-fitting categorical model RSM with the response RSM while partialling out the acoustic RSM, (iv) Bonferroni corrected p-value for the correlation obtained with a permutation test.

Audio S1.
Audio excerpt of a rhythm composed of three time intervals with a 1:1:2 ratio.

Audio S2.
Audio excerpt of a rhythm composed of three time intervals with a 3:3:2 ratio.

Audio S3.
Experimental stimuli.
